# Assessment of corneal substrate biomechanics and its effect on epithelial stem cell maintenance and differentiation

**DOI:** 10.1038/s41467-019-09331-6

**Published:** 2019-04-03

**Authors:** Ricardo M. Gouveia, Guillaume Lepert, Suneel Gupta, Rajiv R. Mohan, Carl Paterson, Che J. Connon

**Affiliations:** 1 Institute of Genetic Medicine, Newcastle University, International Centre for Life, Newcastle-upon-Tyne, NE1 3BZ UK; 2 The Blackett Laboratory, Imperial College London, London, SW7 2BW UK; 3 Harry S. Truman Memorial Veterans Hospital, Columbia, MO 65201 MO USA; 4 College of Veterinary Medicine, University of Missouri, Columbia, MO 65211 MO USA

## Abstract

Whilst demonstrated extensively in vitro, the control of cell behaviour via modulation of substrate compliance in live tissues has not been accomplished to date. Here we propose that stem cells can be regulated solely through in situ modulation of tissue biomechanics. By first establishing, via high-resolution Brillouin spectro-microscopy, that the outer edge (limbus) of live human corneas has a substantially lower bulk modulus compared to their centre, we then demonstrate that this difference is associated with limbal epithelial stem cell (LESC) residence and YAP-dependent mechanotransduction. This phenotype-through-biomechanics correlation is further explored in vivo using a rabbit alkali burn model. Specifically, we show that treating the burnt surface of the cornea with collagenase effectively restores the tissue’s mechanical properties and its capacity to support LESCs through mechanisms involving YAP suppression. Overall, these findings have extended implications for understanding stem cell niche biomechanics and its impact on tissue regeneration.

## Abstract (web summary)

The link between how the stiffness of the cornea affects stem cells is unclear. Here, the authors use Brillouin spectro-microscopy to show that mechanical properties of the cornea affect epithelial stem cells and after injury, treating the cornea with collagenase suppresses YAP activation, assisting in regeneration.

## Introduction

The function of the human cornea is largely dependent on the maintenance of a healthy stratified epithelium, which in turn relies upon a population of stem cells located in its periphery (limbus)^1^. These limbal epithelial stem cells (LESCs) proliferate and differentiate to repopulate the central corneal epithelium, where cells constantly undergo maturation, stratification, and ultimately, shedding from the ocular surface. These events have been shown to be modulated by biochemical and biophysical factors^2,3^. However, the mechanisms underpinning the homoeostatic process of LESC self-renewal and differentiation remain largely unclear^4^. This subject was further complicated by previous suggestions that the limbus is not the only epithelial stem cell niche in the cornea and that corneal renewal is not different from other squamous epithelia^5^, two concepts that have since been robustly refuted^2,4,6^. More recently, a number of studies have shown that the behaviour of LESCs, like other stem cell types^7^, is strongly influenced by their immediate mechanical environment. This notion is supported by the cellular stiffness of LESCs^8^, as well as by the distinct structure^9^, composition^10^, and compliance^11^ of the extracellular matrix (ECM) across the cornea. In particular, the impact of substrate stiffness on corneal epithelial cell attachment and viability^12^, proliferation^13^, and mechanosensing^14^ has been explored in vitro, using biomimetic surfaces with elastic moduli defined after corneal biomechanics, as determined by atomic force microscopy (AFM)^15^. These studies showed that corneal epithelial cells grown on relatively soft substrates are able to retain limbal markers whereas cells cultured on corresponding stiff substrates are disposed to differentiate^13,14,16^. This body of work suggests that, at least in vitro, substrate rigidity regulates LESC phenotype via mechanotransduction pathways involving the yes-associated protein (YAP) transcription factor^14^, and possibly other molecular signals (e.g., FAK/RHOA, ERK1/2, MAL, lamin A/C, and β-catenin)^17^. Yet, the role and relevance of tissue biomechanics on the behaviour of LESCs in vivo is still a matter of contention, in part due to the difficulty in characterising the cells’ native mechanical environment with accuracy and detail on intact tissues. The inability to perform such characterisation is a major restriction to the development of new mechanical therapies (i.e., by creating better synthetic niches or in vivo stem cell manipulation to promote tissue regeneration)^17,18^.

We thus set about a series of experiments to test the hypothesis that substrate stiffness within the native limbal stem cell niche is relevant to stem cell phenotype and wound healing, both in vivo and ex vivo. We start by using Brillouin spectro-microscopy (BSM), a technique based on the interaction of light with spontaneous acoustic phonons in the GHz frequency range, to characterise the mechanical properties of live human corneas in a true non-contact, penetrating (three-dimensional), non-destructive mode (unlike atomic force microscopy, rheology, elastography, or tensile testing methods). Previously, BSM has been used to evaluate mechanical properties of cells and tissues both in vivo^19^ and in vitro^20,21^, including in the cornea at relatively low resolutions^22,23^. Our BSM setup is designed with an original wavefront division adaptive interferometer and a piezoelectric actuator^22^ to extinguish the elastically scattered light, thus allowing the organ-wide, in-depth scanning of whole human corneas at very high resolution and within a time frame compatible with live imaging. Therefore, we use the accuracy of this method to identify critical biomechanical differences between the (softer) limbus and the (stiffer) central cornea, and establish a correlation between tissue biomechanics and corneal epithelial cell phenotype. This data thus supports our hypothesis that epithelial cell differentiation across the corneal surface is controlled by changes in substrate stiffness, via the activation of YAP-dependent mechanotransduction pathways. But more importantly, these results suggest the basis for a pharmacological method to control the phenotype of corneal epithelial cells both in vivo and ex vivo, via the modulation of tissue biomechanics. Specifically, we show that LESC-like phenotype can be promoted in healthy and wounded corneas (for improved wound healing) through the application of a collagenase enzyme, using a formulation currently approved by the United States Food and Drug Administration, European Medicines Agency, and Health Canada for connective tissue softening^24–26^ and already employed therapeutically for treating glaucoma^27^. In this perspective, our study demonstrates the concept of phenotype-through-biomechanical modulation in vivo, a concept that opens new possibilities for creating better cell therapies and medical devices for tissue regeneration.

## Results

### BSM shows transition between softer limbus and stiffer central anterior cornea

**Fig. 1:**
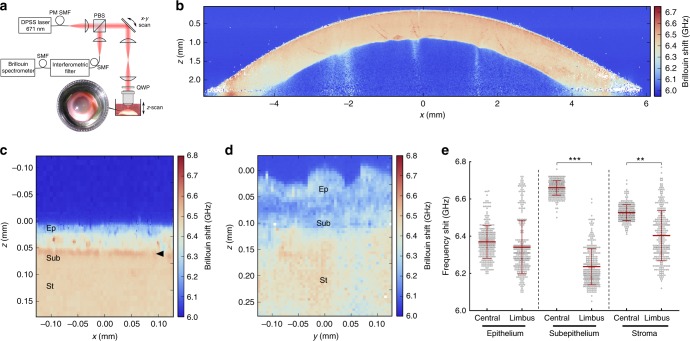
The corneal limbus has distinct mechanic properties. **a** Schematic representation of the Brillouin spectro-microscope (DPSS laser diode-pumped solid-state laser; PBS polarising beam-splitter; QWP quarter-wave plate; PM-SMF polarisation-maintaining single-mode fibre), showing the confocal microscope, elastic scattering filter, VIPA spectrometer, and the sample in its immersion medium. Whole human corneas maintained intact after enucleation were kept immersed in Carry-C to preserve the tissue’s natural thickness, hydration, and transparency state (inset) during Brillouin spectro-microscopy (BSM). **b** Representative organ-wide *X*–*Z* scans of Brillouin frequency shifts from healthy intact human corneas (*n* = 3). Brillouin spectra were acquired with a sample spacing of 20 µm, over a 12 × 3 mm (600 × 150 = 9 × 10^4^ points) *X–Z* transverse section, corresponding to the full corneal width and depth, respectively. These high-resolution scans revealed transversal striæ with high-Brillouin frequency shifts, mostly in the posterior stroma and extending obliquely to the mid or anterior region of the tissue, which probably corresponded to lamellar undulations thought to protect the stromal ultrastructure and shape from external mechanical shocks and the subsequent increase in intraocular pressure^65^. **c** Representative *Y*–*Z* scan of Brillouin frequency shifts of central cornea performed every 2.5 µm, showing a distinct epithelium (Ep; depth = 0–50 µm), sub-epithelial layer (Sub; 50–65 µm), and stroma (St; >65 µm), as well as the location of the Bowman’s layer (arrowhead). **d** Representative *Y*–*Z*-scan of Brillouin frequency shifts of corneal limbus performed every 5 µm, showing the epithelium (depth = 0–50/60 µm), sub-epithelial layer (60–120 µm), and stroma (>120 µm). **e** BSM measurements performed through the anterior region of the central cornea and limbus were statistically analysed (two-way ANOVA; 100 individual measurements per area, per experiment), along with the average (centre line) ± S.D. values (whiskers) from three independent experiments (*n* = 3; **p < 0.01 and ***p < 0.001). Shift value distribution was consistently similar between individual corneas but distinct in central vs*.* limbus

The BSM setup used in this study allowed the whole-organ imaging of immersed human corneas (Fig. 1) to be performed through the entire 12 mm-span and 3 mm-depth of the cornea, at high resolution (Fig. 1b), speed (0.01–1 s per measurement), and accuracy (Supplementary Fig. 1). This approach facilitated the identification of numerous crucial biomechanical features. Firstly, it showed that the limbus had, overall, lower Brillouin frequency shift values, indicating it was more compliant than the central cornea (Fig. 1b). Secondly, it identified the highest frequency shifts (i.e., stiffest tissue) in a discontinuous, 10–15 µm-thick sub-epithelial layer of the central cornea (Fig. 1c), probably corresponding to the Bowman’s layer^11^. Finally, it showed that this layer of stiff matrix was absent in the limbus (Fig. 1d). The substantial differences in Brillouin frequency shift between the anterior region of the central cornea and the limbus prompted a quantitative evaluation of these regions (Fig. 1e). Brillouin transversal section scans in the central cornea showed a multi-layered epithelium ~50 µm thick (Fig. 1c) with average ± S.D. frequency shift of 6.37 ± 0.09 GHz (Fig. 1e) and basal columnar cells presenting stiff nuclei, as previously predicted^14^, followed by a 10–15 µm-thick Bowman’s layer and the anterior-most stroma with 6.66 ± 0.04 and 6.53 ± 0.04 GHz shifts, respectively (Fig. 1e). In contrast, the limbal epithelium ranging 40–60 µm depth (Fig. 1d) and 6.34 ± 0.14 GHz was followed by matrix with significantly lower frequency shift (6.24 ± 0.09 GHz), which in turn was followed by tissue exhibiting slightly higher shifts (6.40 ± 0.14 GHz, respectively; Fig. 1e), probably corresponding to the continuation of the corneal stroma under the limbus (Fig. 1b)^28^.

Consistently, the distinction in frequency shifts between epithelium and the sub-epithelial matrix immediately underneath was less clear in the limbus than in the central cornea (Fig. 1c and d). The limbus also showed a broader distribution range of Brillouin frequency shift values in all its layers, whereas the central corneal was characterised by uniform biomechanical properties within each layer (Fig. 1e), with a profile consistent to that from previous studies^23^. The clear distinction between the mechanically heterogeneous limbus and the more regular central cornea was more evident in very high-resolution scans (Supplementary Figs. 1 and 2) and under alternative vantage points (Supplementary Movies 1 and 2), and consistently observed in all corneas analysed, irrespectively of donors’ gender, or age. This distribution pattern was probably due to the furrowed topography of the limbus, and the presence of numerous structures, such as the Palisades of Vogt, focal stromal projections, and limbal crypts and/or pits (Supplementary Movie 1), particularly abundant in the superior and inferior limbus but less so in the temporal and nasal side of the cornea^2,29–31^ (Supplementary Fig. 2). Moreover, high-resolution Brillouin scans also identified multiple pocket regions within the limbal epithelium that exhibited significantly lower shifts compared to their immediate surrounds (Fig. 1d; Supplementary Fig. 3a). Three-dimensional Brillouin scans showed that these pockets comprise multiple spherical units 10–12 µm in diameter (Supplementary Movie 2) that are compatible, dimension^32^, and biomechanical-wise^8^, with LESCs surrounded by stiffer cells and matrix.

### BSM and immunofluorescence shows LESCs residing on soft limbal matrix

**Fig. 2:**
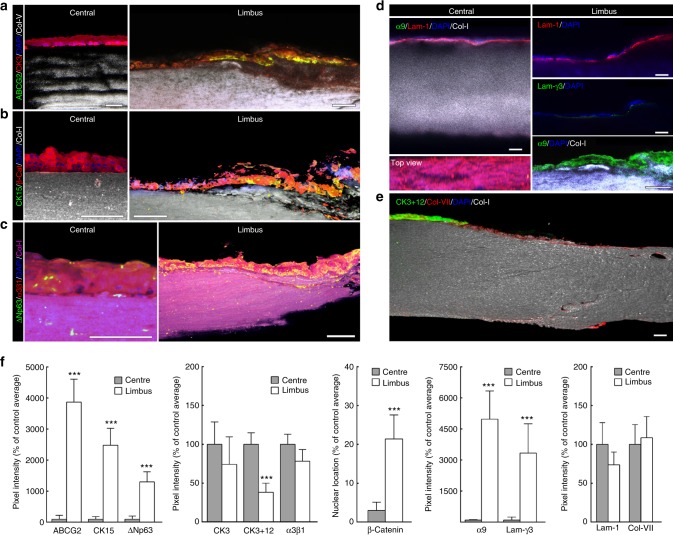
LESC residency corresponds to limbal regions with distinctly softer mechanical properties. Representative confocal immunofluorescence micrographs of corneal epithelial cell markers and ECM components were used to reconstruct in 3D the limbus and central cornea. Expression of limbal markers ABCG2 (**a**), CK15 (**b**), and ΔNp63 (**c**), were expressed by limbal epithelial cells supported by collagen-I-positive/collagen-V-negative matrix, but not by central corneal epithelium (**a**–**c**). Markers such as CK3 (**a**), β-catenin (**b**), and integrin-α3β1 (**c**) showed higher expression in the central corneal epithelium. Histochemical distinction between central cornea and limbus was further evidenced by the basement membrane markers and corresponding receptors. The limbus showed a discontinuous distribution of laminin-1 compared to the central cornea, and the specific expression of laminin-γ3 and integrin-α9 (**d**). Conversely, the central corneal epithelium was positive for CK3+12 and collagen-VII (**e**). Cell nuclei were detected using DAPI. **f** Marker expression was quantified and represented as average ± S.D. from all three independent experiments (*n* = 3; *** corresponds to *p* < 0.001 after one-way ANOVA). Source data are provided as a Source Data file. Scale bars, 100 µm

Importantly, these limbal pockets identified by BSM also represented the location of cells co-expressing ABCG2, CK15, nuclear β-catenin, and ΔNp63 (Fig. 2a–c; Supplementary Fig. 3b), markers consensually associated with LESCs^33–35^. In contrast, the CK3/12-positive epithelial cells from the central cornea were negative for limbal markers (Fig. 2a–c), indicating that these constituted a more differentiated epithelium. The co-location of areas of low-Brillouin shift with the immuno-anatomical limbus was further confirmed by the geometry of its features (Supplementary Fig. 3), as well as by the expression of ECM components and corresponding cell receptors (Fig. 2). The distribution of the low-Brillouin shift within limbal sub-epithelial matrix (Fig.1e), classically identified as conjunctival stroma and Tenon’s capsule^28^, was well correlated to the collagen-I-positive/collagen-V-negative connective tissue immediately underlying the resident LESCs (Fig. 2a–c). Moreover, the focal distribution of laminin-γ3, a characteristic marker of limbal basement membrane^36^ along with the strong expression of integrin-α9, a transient amplifying cell/LESC marker^37^, showed that this region corresponded to the corneal limbus (Fig. 2d). The low-Brillouin shift values were also well correlated with the tissue’s structural features, previously characterised as comprising less compact collagen lamellae with irregularly arranged, branched, and intertwined collagen bundles^38,39^. In contrast, the location of CK3-positive epithelial cells (Fig. 2e) was well correlated to the regions of the central cornea with the highest-Brillouin shift values (Fig. 1b). These regions showed to be comprised by ubiquitous corneal or conjunctival basement membrane components, such as collagen-VII^10^ and laminin-1^36^, but not laminin-γ3 (Fig. 2d, e). Furthermore, the matrix under the central corneal epithelium was strongly positive for both collagen-I and collagen-V (Fig. 2). This distinctive composition plays an important role on collagen fibril diameter and lamellar organisation of the corneal stoma^40^, which in turn has a critical influence in tissue transparency and elastic modulus^9,41^. Signal quantification also supported the distinctive expression pattern of markers in both limbus and central cornea (Fig. 2f). Taken together, these results showed that, in the human cornea, LESCs populate tissues that are substantially softer compared to those supporting differentiated epithelial cells, thus constituting a niche with distinct biomechanical, as well as a biochemical/biomolecular profiles.

### Substrate stiffness controls corneal epithelial cell phenotype in vitro

The surprisingly distinct properties between central cornea and the limbus observed by BSM reinforced our hypothesis that biomechanics plays a role in controlling the phenotype of corneal epithelial cells^14^. The stiffer features of the central cornea support the notion that a biomechanical differential (i.e., gradient or step-change in substrate stiffness) plays a crucial role on corneal epithelial cell differentiation via mechanotransduction^14,42^. Indeed, several pathologies compromising corneal integrity are currently managed by collagen cross-linking, where the stiffening of the central cornea^23^ (but not of the limbus) allows the restoration of a healthy differentiated epithelium^43^. Conversely, in the limbus, cells residing in the basal epithelial layer predominantly express the inactivated form of YAP, an indication of reduced exposure to mechanical inputs^14^. Moreover, aberrant stiffening of the limbus (e.g. due to fibrosis) is associated with pathologies leading to stem cell loss^44^. Assuming that the differences in tissue modulus (between limbus and central cornea) reported here are vital for corneal function/homoeostasis, then it is reasonable to consider the manipulation of substrate stiffness as an effective therapeutic approach to promote/restore LESC function in damaged corneas. We thus developed a strategy to investigate the response of LESCs to surface compliance, first using an in vitro model.

**Fig. 3:**
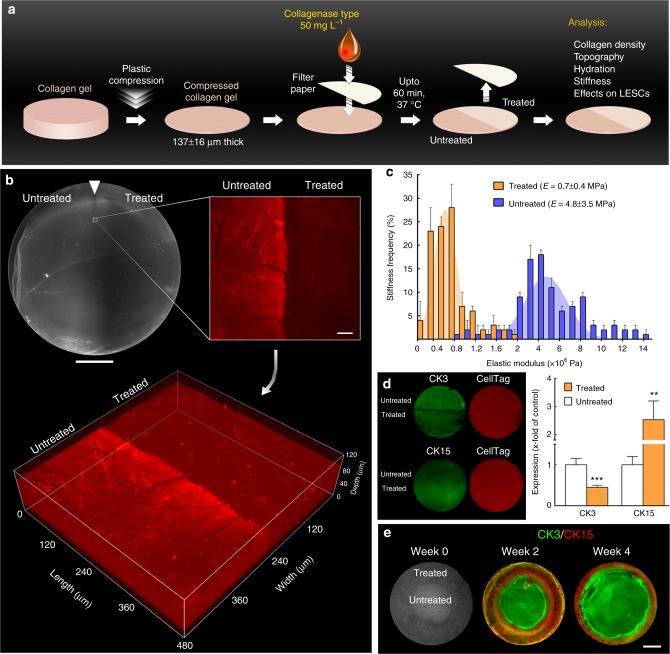
LESC-like phenotype can be controlled through fine modulation of the mechanical properties of collagen substrates. **a** Schematic representation of the collagenase treatment method used to modulate the stiffness of compressed collagen gels. High-density, plastic-compressed collagen gels were softened with collagenase type-I solution in well-defined areas (ring-shaped, semi-circular, or entire gel surface) for up to 60 min. **b** Analysis of compressed collagen gel density after collagenase treatment. The regions corresponding to collagenase-treated collagen gels showed increased transparency under bright-field imaging (upper left panel; scale bar, 5 mm) and lower collagen density compared to untreated regions, as indicated by the lower collagen-I detection by immunofluorescence confocal microscopy (upper right panel; scale bar, 50 µm). The confocal *Z*-scans (lower panel) demonstrated that the difference in signal intensity between treated and untreated areas was not restricted to the surface, indicating that the collagenase in solution acted through the entire depth of the compressed collagen maintaining a defined treatment zone. **c** Average frequency ± S.D. of the elastic modulus, *E* (MPa), of treated (orange) and untreated gels (blue bars) calculated from three independent experiments using force–distance spectroscopy (*n* = 3). The frequency histograms of treated and untreated gels were used to calculate Gaussian curves by non-linear regression (orange and blue areas, respectively), with corresponding *E* = 0.7 ± 0.4 and 4.8 ± 3.5 MPa. **d** Effects of substrate stiffness on the expression of CK3 (differentiation) and CK15 (LESC protein marker) in cells grown for 4 weeks on treated and untreated regions of collagen gels (green staining) after normalisation for total cell number (red staining), and represented as average ± S.D. from three independent experiments (*n* = 3; ** and *** corresponds to *p* *<* 0.01 and 0.001 after one-way ANOVA, respectively). Source data are provided as a Source Data file. **e** Creation of a pseudo-limbus. Cells growing on ring-shaped softened areas (week 0) expressed higher levels of CK15 compared to the high CK3-positive cells growing on the untreated (stiffer) central region of the collagen gels, up to 4 weeks in culture (scale bar, 5 mm)

Previously, high-density collagen-I gels (tissue mimics) with different levels of stiffness via plastic compression were used to that purpose^45,13^. Now we explored the premise that collagen gel stiffness could be modulated locally with topical applications of a type-I collagenase solution (Fig. 3a). Collagen gels treated for up to 60 min were shown to be partially digested, with enzymatic cleavage restricted to the collagenase-soaked areas (Fig. 3b, treated vs. untreated), and acting through the entire thickness of the matrix without reducing gel thickness (Fig. 3b) or compromising its structural integrity (Supplementary Fig. 4a–c). Specifically, collagen fibril morphology was indistinguishable between treated and untreated gels (Supplementary Fig. 4b, c) and comparable to that in the literature^46^. Moreover, this partial digestion did not affect the gels’ average surface roughness (Supplementary Fig. 4c), thus presenting nano-topographical features substantially flatter than those previously shown to affect LESC phenotype^47,48^. However, collagenase greatly reduced gel density, with a 33 ± 16% dry weight loss and increased 83 ± 4% hydration after 60 min treatment (Supplementary Fig. 4d), indicating that the enzyme was able to penetrate through the compressed gels despite being applied topically. The lower collagen bulk and higher gel hydration as a function of treatment duration also corresponded to significant reductions in elastic modulus (Supplementary Fig. 4e, f), with gels treated for 60 min presenting an average *E* = 0.7 ± 0.1 MPa, almost an order of magnitude lower compared with mock-treated gels (*E* = 5.2 ± 1.2 MPa) (Fig. 3c).

This difference in gel compliance constituted an important result, as it was subsequently shown to affect corneal epithelial cell phenotype. LESCs isolated from human corneal tissues and grown on softened (treated) collagen gels showed a significantly increased CK15 (limbal stem/progenitor cell marker) and decreased CK3 (differentiated corneal epithelium marker) expression at both transcriptional and protein levels (Fig. 3d; Supplementary Fig. 5a), in a well-defined pattern delimited by the boundaries of enzyme treatment (Fig. 3d), and without induced expression of pro-inflammatory factors^49–51^ (Supplementary Fig. 5b). Importantly, cells on collagenase-treated gels also showed a significant reduction in YAP, both in terms of total expression and nuclear location (nYAP) (Supplementary Fig. 5c), supporting previous evidence that softer substrates promote LESC phenotype via key YAP-dependent mechanotransduction signalling pathways^14^. Moreover, these cells maintained LESC-characteristic morphologies, with a diameter of 10–12 µm and high nucleus-to-cytoplasm ratio (Supplementary Fig. 5c). In contrast, cell morphology on untreated gels was closer to that of basal epithelial cells from the centre of the cornea^32^.

These results indicated that the enzymatic digestion can produce precisely controlled, localised differences in the stiffness of high-density collagen gels, resulting in the removal of up to one-third of the original collagen content from within the gels without altering their volume, nano-structure^46^, or nano-topography^48^, and subsequently maintain undifferentiated LESCs. As such, we then used the collagenase treatment to create a ring of softer collagen (representing the limbus, having shown by BSM that this tissue is significantly softer) surrounding a stiffer area (representing the central cornea). Cells subsequently grown on the treated, outer-ring area (pseudo-limbus) expressed higher levels of CK15, whereas cells on the central, untreated region of the gels displayed higher CK3 expression (Fig. 3e). The ratio between CK3-positive and CK15-positive cells increased after 4 weeks in culture, mainly due to increased cell stratification over time. However, the maintenance of CK15 expression by LESCs on collagen gels was, overall, substantially higher than on infinite-stiffness substrates (i.e. tissue culture plastic) at equivalent stages in culture (Supplementary Fig. 6). Moreover, these effects were independent of the presence of any potential cryptic epitopes exposed by collagenase treatment (Supplementary Fig. 7).

### Substrate stiffness controls corneal epithelial cell phenotype ex vivo

**Fig. 4:**
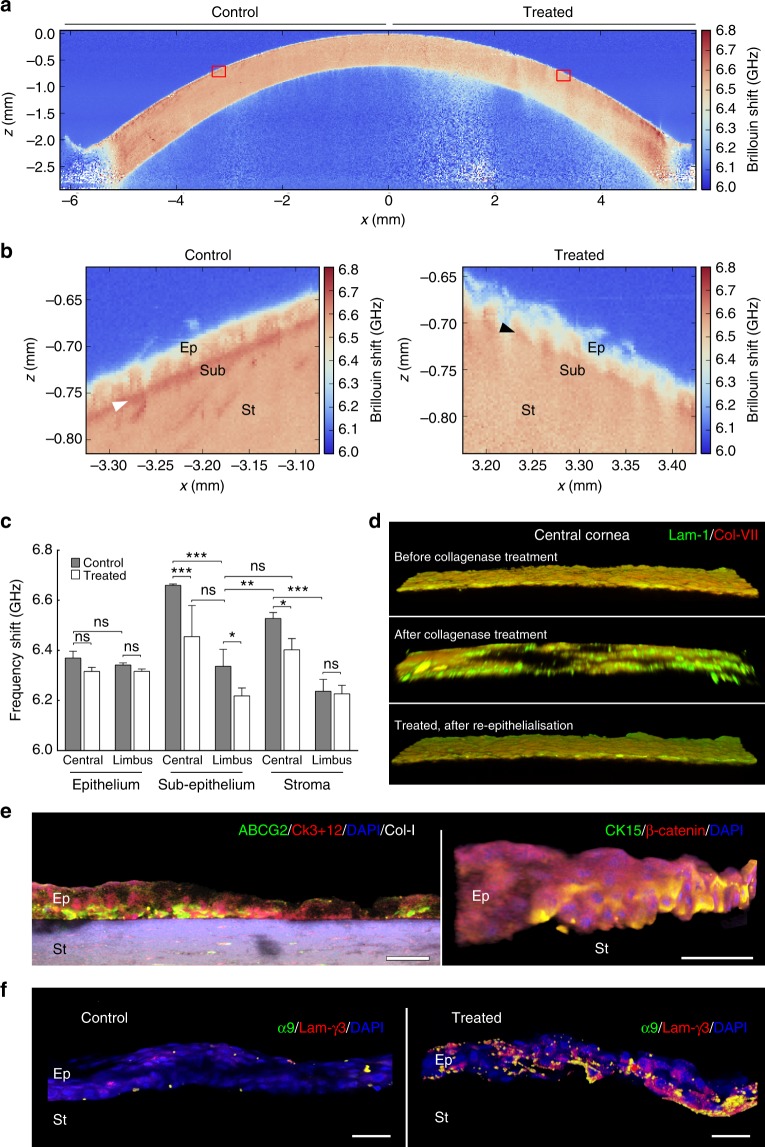
Softening of corneal tissue with collagenase increases expression of limbal markers during ex vivo re-epithelialisation. **a** Representative whole-cornea *X*–*Z* scan of Brillouin frequency shifts measured at a sample spacing of 20 µm from healthy intact human corneas after collagenase treatment (*n* = 3). Insets correspond to (**b**) the regions of the central cornea softened with collagenase (treated) or left untreated (control) analysed at very high-resolution scanning, using a sampling distance of 2.5 µm, showing a distinct epithelium (Ep), sub-epithelial layer (Sub), and stroma (St), as well as the location of the Bowman’s layer (white arrowhead). This detailed analysis evidenced the loss of the stiffer Bowman’s layer (black arrowhead) and reduced Brillouin frequency shifts in both epithelium and stroma resulting from collagenase treatment. **c** Brillouin frequency shifts from the anterior part of both treated (white) and control regions (grey bars) of the central cornea and limbus. The plot represents average ± S.D. of measurements taken from the epithelium, sub-epithelium, and stroma from three independent experiments (100 individual measurements per each individual area, per experiment; *n* = 3; ns corresponds to *p* > 0.05 and *, **, and *** to *p* < 0.05, 0.01, and 0.001 after two-way ANOVA, respectively). Source data are provided as a Source Data file. **d** Representative confocal immunofluorescence micrographs (3D reconstruction) of laminin-1 (green) and collagen-VII distribution (red staining) in the central cornea, before (upper) and after collagenase treatment (central panel), and after re-epithelialisation (lower panel). **e** Representative confocal immunofluorescence micrographs (3D reconstruction) of collagenase-softened central cornea after re-epithelialisation. Epithelial cells repopulating softened corneas expressed ABCG2 and CK15 (limbal markers; green) while showing lower CK3+12 and β-catenin expression (differentiation markers; red staining) compared to cells growing on the stiffer, untreated corneas (control). **f** Epithelial cells expressed integrin-α9 and deposited laminin-γ3 when grown on collagenase-softened corneal substrates, but not on untreated central cornea (control). Cell nuclei were detected using DAPI in all three independent experiments. Scale bars, 50 µm

Based on these in vitro results, the application of collagenase could represent an effective and safe treatment to help restore appropriate levels of tissue compliance to a diseased (stiffer) limbus, and thus promote LESC maintenance and function. Collagenase could also be used on the central cornea to alter the mechanical properties of its matrix towards obtaining a more compliant, limbus-like environment. In other words, collagenase could be used to extend or even restructure a ‘new’ limbus within the periphery of the cornea, and thus provide resident or transplanted LESCs with a suitable alternative mechanical niche. To test this possibility, we applied collagenase ex vivo to well-delimited central areas of freshly enucleated human corneas (Supplementary Fig. 8) and analysed the softening effect by BSM (Fig. 4a). As predicted from the plastically compressed collagen gel study, topical collagenase application to the human cornea resulted in lower Brillouin frequency shifts in the collagenase-treated, but not in mock-treated areas of the cornea (Fig. 4a), and with no loss of accuracy (Supplementary Fig. 8b). Specifically, the collagenase treatment significantly lowered the Brillouin frequency shift in the central sub-epithelial and anterior stroma matrix compared to controls (Fig. 4b) while surprisingly maintaining the epithelium layers in place, albeit in a less evenly distributed pattern (Fig. 4b, right panel). Notably, the Brillouin frequency shift from the treated sub-epithelial matrix of the central cornea (6.45 ± 0.13 GHz) was statistically similar and presented a similar variation to that of the sub-epithelium from the control (untreated) limbus (6.34 ± 0.07 GHz) (Fig. 4c). This indicated that collagenase treatments can soften the central cornea towards a limbus-like level of compliance.

The altered mechanical properties of the collagenase-treated central cornea were in part derived from the degradation of the collagen-rich matrix of the anterior stroma, including the Bowman’s layer (Fig. 4b). However, collagenase was equally effective in softening the normal corneal limbus (where the Bowman’s layer is absent), with localised ex vivo treatments significantly reducing matrix stiffness (Supplementary Fig. 8c), namely at the sub-epithelium level (Fig. 4c). Confocal immunofluorescence analysis showed that the basement membrane in the central cornea was also affected, presenting large gaps in the distribution of collagen-VII and laminin-1 following collagenase treatment (Fig. 4d). Nonetheless, after careful debridement through washing, both treated and control central corneas successfully served as ex vivo growth substrate for LESCs, supporting re-epithelialisation (Fig. 4e) and basement membrane re-deposition (Fig. 4d). Similarly to their behaviour on collagen gels, cells grown on softened areas of the central cornea continued to express limbal markers, such as CK15 and ABCG2, particularly at the basal layers of the new epithelium (Fig. 4e). Furthermore, they deposited focalised laminin-γ3 and expressed integrin-α9 (Fig. 4f), two markers characteristic of the limbal niche^36,37^. In contrast, cells grown on mock-treated areas of the central cornea failed to express any of these markers. Signal quantification showed that this differential expression was significant for all markers tested (Supplementary Fig. 9).

### Substrate stiffness controls corneal epithelial cell phenotype in vivo

**Fig. 5:**
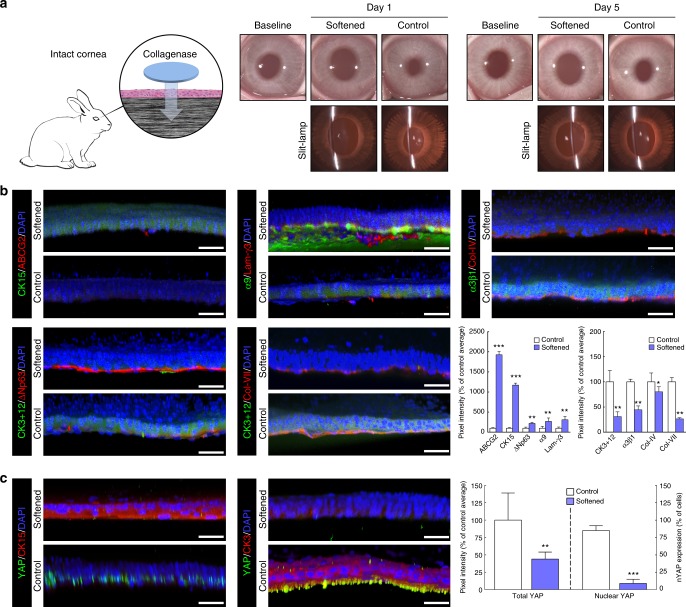
Softening of central corneal tissue with collagenase increases expression of limbal markers in vivo. **a** Schematic representation of the collagenase treatment method used to soften the central region of intact corneas in live rabbits. Clinical observation and slit-lamp examination was performed 1 and 5 days post-intervention, and compared to pre-intervention results (baseline). The central corneal epithelium was also analysed by confocal immunofluorescence (3D reconstruction) 5 days after collagenase treatment (softened), and the corresponding marker expression was quantified. Cells on softened corneas expressed (**b**) higher levels of ABCG2, CK15, ΔNp63, integrin-α9, and laminin-γ3 (limbal markers) and lower levels of CK3+12 and integrin-α3β1 (differentiation markers) compared to cells growing on the stiffer, untreated corneas (control). Collagen-IV and collagen-VII were also detected in collagenase-treated (softened) corneas, albeit at lower levels compared to control. **c** The expression of the mechanotransduction marker YAP was also significantly lower in the CK15-positive cells on softened corneas, where it mostly presented a non-nuclear (inactive) form compared to that in CK3-positive cells on untreated corneas (control). Cell nuclei were detected using DAPI. Scale bars, 50 µm. Marker expression was represented as average ± S.D. from three independent experiments (*n* = 3; *, **, and *** corresponds to *p* < 0.05, 0.01, and 0.001 after one-way ANOVA, respectively). Source data are provided as a Source Data file

Next, we investigated the effects of substrate stiffness on corneal epithelial cells in vivo, using collagenase to soften the central region of rabbit corneas (Fig. 5). The outcome of collagenase treatment on intact rabbit corneas (i.e. without prior debridement) was followed by clinical observation, slit-lamp biomicroscopy, and immunohistochemistry analyses 1 and 5 days post-intervention (Fig. 5a). Interestingly, the in vivo softening elicited a radical change in epithelial cell phenotype, with molecular markers showing major differences in expression compared to corresponding controls (mock-treated rabbit corneas; Fig. 5b), in line with the effects obtained in vitro (with collagen gels) and ex vivo (with organ-cultured human corneas). Specifically, epithelial cells residing on the softened centre of rabbit corneas were shown to express CK15, ABCG2, ΔNp63, laminin-γ3, and integrin-α9 5 days after treatment (Fig. 5b). Conversely, softening the central cornea induced a reduction in CK3/12 expression in the epithelium and resulted in a partially degraded basement membrane, as shown by the reduced levels of collagen-IV and collagen-VII (Fig. 5b). In addition, the mechanotransduction factor YAP changed drastically in softened corneas, with CK15-positive cells showing reduced YAP expression and loss of its active (nuclear) form (nYAP) compared to that in CK3-positive cells from mock-treated corneas (Fig. 5c). These differences in expression were significant (Fig. 5b, c, graphs), and represented important alterations in the normal phenotype of epithelial cells from the central cornea. In particular, the significant reduction (*p* = 0.0002) of the active nYAP in the basal epithelium following collagenase treatment demonstrated that softer substrates indeed promoted YAP inactivation, and further supported the notion of YAP-mediated mechanotransduction playing a crucial role in defining LESC phenotype in vivo. Moreover, treatments maintained corneal integrity and transparency (Fig. 5a), and failed to produce any substantial irritation, inflammation, oedema, or change in intraocular pressure (IOP) (Supplementary Table 1). Furthermore, no conspicuous neo-vascularisation was observed following collagenase treatment of the cornea either on a macroscopic (Fig. 5a) or microscopic level (Supplementary Fig. 10). The maintenance of an intact epithelium and its corresponding barrier function, even after collagenase treatment, was probably the main factor protecting the corneas from these deleterious effects (Fig. 5a).

### Collagenase treatment restores limbus capacity to support LESCs after alkali burn

**Fig. 6:**
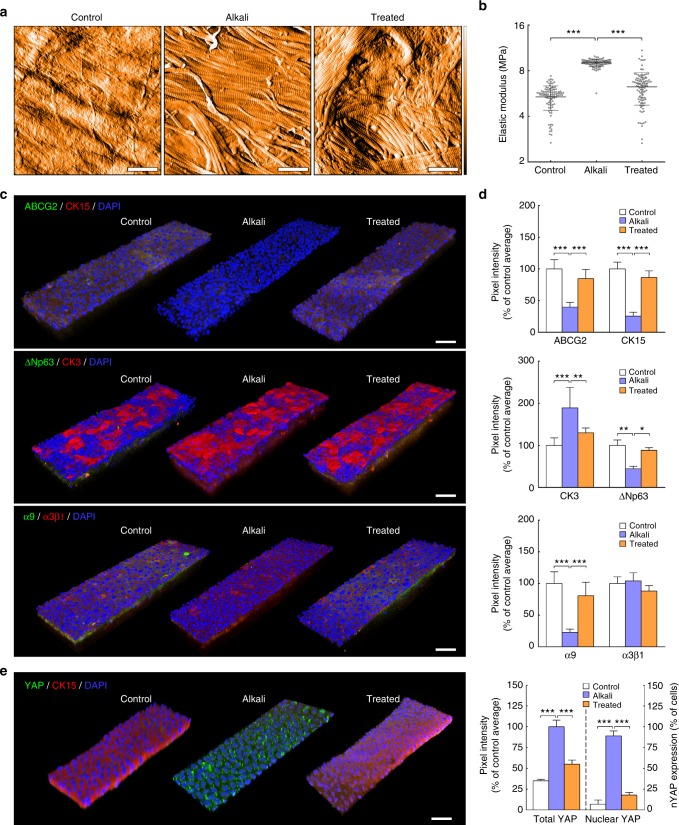
Softening of alkali-burned corneal tissue with collagenase restores expression of limbal markers ex vivo. **a** Representative topography of limbal sub-epithelial matrix after application of PBS (control), 0.5 M NaOH (alkali), or NaOH followed by collagenase softening (treated) of whole human corneas, analysed by atomic force microscopy (AFM). AFM scans from three independent experiments showed clearance of ECM components other than collagen fibrils in tissue subjected to alkali burn, and reduced collagen fibril density after collagenase treatment. False colour depth, 500 nm. **b** Force–distance spectroscopy analysis showed that the burn (alkali) significantly stiffened the limbal sub-epithelial matrix, and that subsequent collagenase softening (treated) restored the mechanical properties of the original tissue (control). The graph represents the distribution of calculated values of elastic modulus, *E* (MPa), and corresponding average (centre line) ± S.D. (whiskers) from three independent experiments (*n* = 3; ***p < 0.001). **c** Representative confocal immunofluorescence micrographs (3D reconstruction) of re-epithelialised limbus after alkali burn and collagenase treatment, with (**d**) corresponding marker expression quantification. Epithelial cells repopulating the alkali-burned limbal tissue ex vivo expressed significantly lower levels of limbal markers ABCG2, ΔNp63, CK15, and integrin-α9 while showing higher expression of CK3 differentiation markers compared to cells growing on control tissue. **e** The mechanotransduction marker YAP also changed significantly in tissues made stiffer by alkali, with repopulating cells showing increased YAP expression and a predominant nuclear localisation. However, collagenase treatment successfully restored the ability of burned limbal tissues to support cells expressing LESC, differentiation, and mechanotransduction markers at control levels. Cell nuclei were detected using DAPI. Marker expression was represented as average ± S.D. from three independent experiments (*n* = 3; *, **, and *** corresponds to *p* < 0.05, 0.01, and 0.001 after one-way ANOVA, respectively). Source data are provided as a Source Data file. Scale bars, 1 µm (**a**), 50 µm (**c**, **e**)

Finally, to further explore the potential clinical relevance of modulating tissue biomechanics, collagenase was used to soften the artificially stiffened limbus in alkali-burned corneas. This type of injury was chosen for its extensive prior use to model corneal pathologies causing corneal fibrosis and stiffening, as well as LESC deficiency (LSCD) and loss. In the first of these preliminary experiments, human corneas subjected to alkali burns ex vivo were treated with collagenase and tested for their mechanical properties, as well as their ability to support cells with a LESC-like phenotype (Fig. 6). As expected, the alkali burn led to major alterations in the corneal limbus, with clearance of most of its non-fibrillar elements and the significant, localised, and consistent stiffening of its matrix (Fig. 6a and b). These effects were probably due to proteoglycan removal following alkali exposure^52^. Subsequently, alkali-burned corneas also failed to maintain the LESC-like phenotype of repopulating cells. Specifically, LESCs seeded on the limbus of alkali-burned corneas showed significantly reduced expression of limbal markers ABCG2, CK15, ΔNp63, and integrin-α9, while expressing higher levels of differentiation marker CK3 compared to cells on non-burned, control tissue (Fig. 6c and d). In addition, cells on the burned limbus showed a significant increase in YAP expression and nuclear localisation (*p* = 0.0003 and 0.0002, respectively) compared to those on control tissues (Fig. 6e), suggesting that phenotype loss was driven by YAP-dependent mechanotransduction signalling. However, all deleterious effects were shown to be reversed by application of collagenase on alkali-burned corneas, with treatment leading to reduced collagen fibril density and significant decrease in the stiffness of the limbus matrix (Fig. 6a and b). But most importantly, the recovery of the original tissue compliance was accompanied by the ability to support re-epithelialisation by cells expressing limbal stem/progenitor cell markers (Fig. 6c and d). Furthermore, and as expected from our preceding experiments, this response was accompanied by a significant reduction in YAP expression and nuclear localisation (Fig. 6e).

**Fig. 7:**
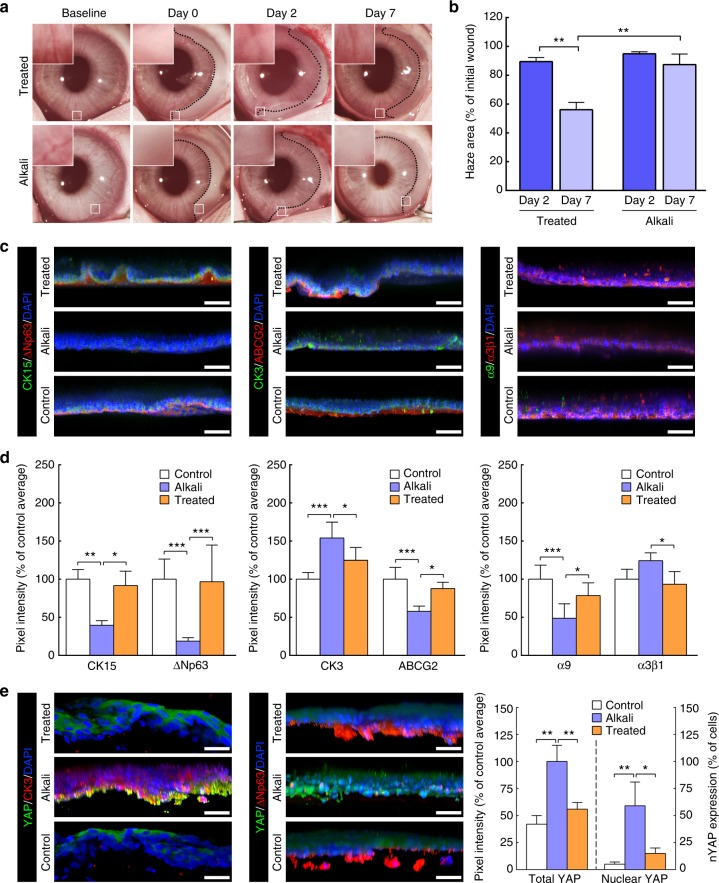
Treatment of alkali-burned limbus with collagenase restores expression of limbal markers in vivo. **a** Representative images of chemically burned rabbit corneas (alkali) and of alkali-burned corneas receiving collagenase treatment (treated), before (baseline) and after burn (day 0), as well as at day 2 and 7 post-burn. The haze resulting from the burn was delimited (traced line), and analysed for recovery (insets), and covered area (**b**) at day 0 (initial wound), 2 (dark blue) and 7 (light blue bars). Values corresponded to haze area average ± S.D. from three independent experiments (*n* = 3). **c** Representative confocal immunofluorescence micrographs (3D reconstruction) of chemically burned (alkali) and collagenase-treated burned limbus (treated) at day 7, with (**d**) corresponding marker expression quantification. Cell nuclei were detected using DAPI. Epithelial cells repopulating the alkali-burned limbus in vivo expressed significantly lower levels of limbal markers CK15, ΔNp63, ABCG2, and integrin-α9 while showing higher expression of the CK3 differentiation marker compared to the undamaged limbus (control) tissue. However, collagenase treatment successfully restored the ability of the burned limbus to support cells as in control tissues. **e** The expression of the mechanotransduction marker YAP was also significantly lower in the ΔNp63-positive, CK3-negative cells on treated and control limbus tissues, where it predominantly presented a non-nuclear, inactive form. In contrast, CK3-positive, ΔNp63-negative cells on the alkali-burned tissues showed significantly higher, and mostly nuclear, YAP expression. The expression of markers in **d**, **e** was represented as average ± S.D. from three independent experiments (*n* = 3; *, **, and *** corresponds to *p* < 0.05, 0.01, and 0.001 after one-way ANOVA, respectively). Source data are provided as a Source Data file. Scale bars, 50 µm

Similar results were observed in a second set of experiments, this time using an in vivo chemical burn model. Rabbit corneas subjected to a localised alkali burn had the temporal half of the limbus damaged while maintaining its nasal half intact, to allow re-epithelialisation (Supplementary Fig. 11, day 0). On the second day post-burn, the affected areas of the limbus were either treated with collagenase (treated) or remained untreated (alkali) (Supplementary Table 2), and were subsequently compared to undamaged limbal tissue (control). Immediately after the burn, the affected area of the limbus became opaque (Fig. 7a). In untreated corneas, this haze showed little or no reduction in size (Fig. 7b). In contrast, collagenase-treated tissues showed a significant reduction of haze area compared with alkali-burn tissues (*p* = 0.0023) and with time (90 ± 3–56 ± 5% of initial size from day 2 to 7, respectively; *p* = 0.002) (Fig. 7b), probably through restoration of epithelial integrity and barrier function (Supplementary Fig. 12). Furthermore, treating the burned limbus with collagenase allowed the tissue’s native cellular profile to be restored (Fig. 7c). Specifically, the new epithelium populating the collagenase-treated burned limbus tissue showed significantly higher expression of limbal markers CK15, ΔNp63, ABCG2, and integrin-α9, and lower expression of differentiation markers CK3 and α3β1 compared with their untreated (alkali) tissue counterparts, and an expression profile similar to the undamaged (control) limbus (Fig. 7c and d). These cells probably originated from the neighbouring nasal limbus kept intact during the challenge. Furthermore, the reduction of haze during the healing process (Fig. 7a) and the absence of goblet cells in the new epithelium (Fig. 7c; Supplementary Fig. 12) suggested that the repair was not made by transdifferentiating conjunctival cells. Alternatively, the new epithelium might have derived from dedifferentiation of corneal committed cells, as recently shown to occur in mice after surgical deletion of LESCs, but not after alkali burn of the limbus^6^.

Interestingly, YAP expression in cells repopulating collagenase-treated tissues was similar to that on the undamaged limbus, and significantly (*p* = 0.0015) lower to that on tissues made stiffer by the alkali burn (Fig. 7e). Cells growing on treated tissues also presented significantly (*p* = 0.019) lower levels of nuclear YAP compared to those on alkali tissues, namely in basal layers of the epithelium (Fig. 7e), where ΔNp63 showed particularly strong expression. Moreover, and as previously observed in collagen gels and the central cornea, the variations in YAP expression in the limbus were independent from substrate topography, with application of alkali and/or collagenase showing no evident alterations in limbal nano- or micro-topographical features (Figs. 6 and 7, respectively).

## Discussion

**Fig. 8:**
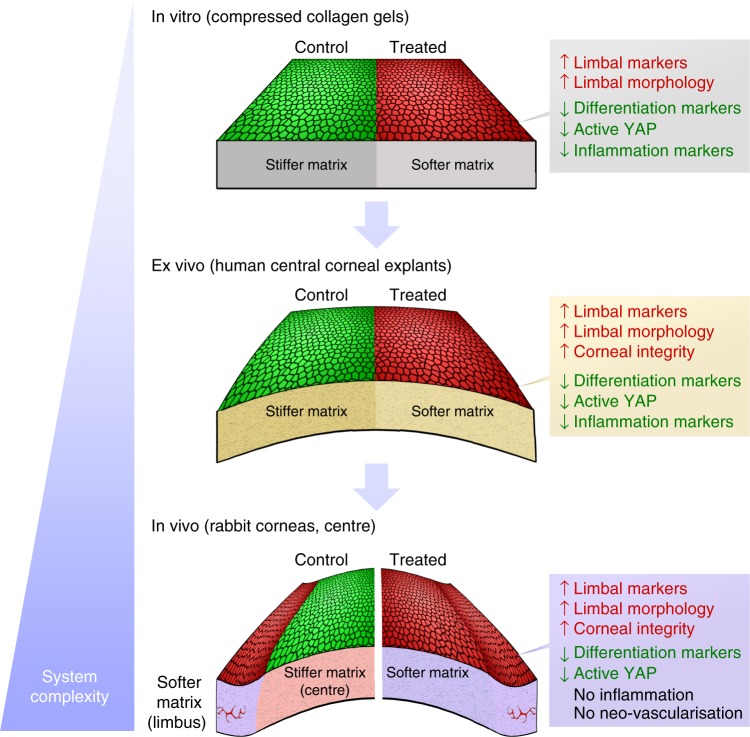
Consistency of overall results from the multiple experimental model systems under study. Epithelial cells (re)populating collagenase-softened (treated) substrates expressed significantly higher levels of limbal markers and lower levels of differentiation and active mechanotransduction markers compared to the untreated (control) tissues, independently of the system’s degree of complexity (i.e., topography, biochemical composition). In addition, the maintenance of limbal cell morphology and failure to elicit major pro-inflammatory responses after tissue softening were similarly observed for all in vitro, ex vivo, and in vivo experiments. The comparable results obtained on both simple and complex substrates supported the notion that the cellular effects were derived from the modulation of tissue biomechanics, and not due to tissue topography or composition (i.e., exposure to cryptic biochemical cues or growth factors)

Overall, our findings demonstrate that the phenotype of human corneal LESCs is highly dependent upon the mechanical properties of their substrate, with matrices reproducing the more compliant biomechanics of the limbus (as detected by BSM) also being able to promote its biological function. Subsequently, we showed that the corneal epithelial cell phenotype can be controlled through modulating the mechanical properties of collagen-rich substrates, both natural (i.e., the cornea) and manufactured (i.e., compressed collagen gels). In particular, the collagenase type-I formulation used in this study provided a simple but efficient method to soften such substrates to reproduce the compliance of the limbus, and thus (re)create its biological function as a niche for epithelial stem/progenitor cell maintenance. This was illustrated in vitro in our pseudo-limbus experiments, where cells residing on collagen gels treated with collagenase retained an undifferentiated, LESC-like phenotype whilst cells on stiffer, untreated collagen gels (pseudo-central cornea) assumed a differentiated phenotype. This modulation of cell behaviour depended on the substrates’ biomechanical similarity with the natural limbus, and not on their structural complexity (e.g., tissue topography or geometry) and/or compositional environment (e.g., interactions with new biochemical cues created after collagenase treatment), as suggested by the mechanosensitive regulation of YAP expression shown in this study (Fig. 8) and in previous work using LESCs on semi-compressed collagen gels^14^. Both collagenase-treated and semi-compressed gels have comparable topographies, moduli, and phenotype-modulating properties; however, the latter are produced by variations in time and load of plastic compression^13^, and therefore their ability to maintain undifferentiated LESCs cannot be attributed to newly exposed cryptic epitopes or changes in topography.

But most notably, the presently developed collagenase treatment represents a potentially valuable off-label^27^ therapeutic strategy to regulate cell phenotype via manipulation of tissue biomechanics, a methodology that can greatly expand the field of mechanical therapy^18^. Using this enzyme to soften the matrix of the anterior layers of the central cornea allowed the (re)creation of a limbus-like mechanical and phenotypic milieu, both in vivo and ex vivo, without compromising the tissue’s integrity, or causing any measurable inflammatory or neo-vascularisation responses (Fig. 8). Crucially, this can have important and extensive applications, namely as a non-hormonal therapeutic method to soften pathologically hardened natural tissues, such as scared corneas. Previous studies reported that alterations of the mechanical properties of the corneal limbus, namely involving its stiffening, can result in LSCD^47^, metaplasia^44^, and consequently to vision loss^53,54^. Furthermore, a damaged limbal microenvironment can limit the use of therapeutic procedures based on LESC transplantation, as the newly transplanted cells are potentially negatively affected by a still-compromised substrate in the post-injury cornea, or by the improper mechanical properties of the graft. Such notion is supported by evidence that LESCs cultured on stiffer, polystyrene substrates without feeder layers show a progressive phenotype change^55^. Furthermore, the LESC phenotype has been shown to be reduced when grown upon human amniotic membrane made stiffer after UV treatment^12^ or debridement^56^. In contrast, LESCs used therapeutically to treat LSCD are currently expanded ex vivo on fibrin gels^57^, a substrate whose elastic modulus of 0.3–0.5 MPa falls within the range measured for our collagenase-treated (softened) collagen gels. These cases provide further evidence that substrate stiffness plays a fundamental role in maintaining LESC phenotype, and that the ability to predict and control the mechanical properties of a substrate may lead to better therapeutic outcomes.

**Fig. 9:**
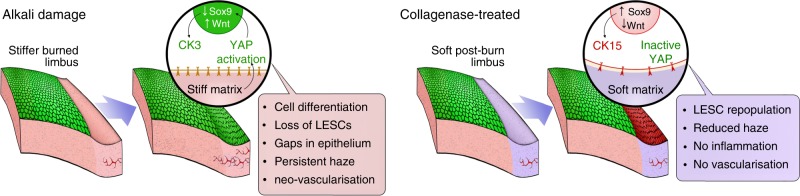
Schematic representation of overall results from in vivo experiments and proposed mechanism of action. Briefly, stiffening of the limbus caused by alkali burn induces the activation and nuclear translocation of YAP, a key regulator of mechanotransduction. The nuclear YAP then acts as a transcription factor, promoting Wtn/β-catenin and suppressing Sox9 signalling, which in turn induce cell differentiation and lead to loss of LESCs. By softening the burned limbus with collagenase, repopulating stem/progenitor cells retain a mostly inactive YAP, with corresponding high Sox9 and low Wtn/β-catenin levels promoting LESC phenotype maintenance

Our animal experiments suggested that collagenase can also be used in vivo to restore a more suitable mechanical environment for endogenous LESCs in alkali-burned corneas. Specifically, we demonstrated that collagenase effectively reversed the stiffening of the corneal limbus following alkali burn, preventing the consequent activation of cellular YAP, and allowing the recovery of its natural LESC-supporting capacity. These findings further supported the role of YAP as a key molecular mediator of corneal mechanotransduction pathways, with increased stiffness of the limbus leading to YAP’s increased nuclear translocation, activation, induced cell differentiation, and subsequent loss of LESC phenotype (Fig. 9, alkali). Conversely, restoring the mechanical properties of the natural limbus (i.e., by using collagenase to soften its matrix) prevented YAP activation and contributed to LESC maintenance and epithelial recovery (Fig. 9, treated). This modulation of cell phenotype probably occurs via the regulation of YAP-dependent transcription factors Wnt/β-catenin^58^ and Sox9^59^ (Fig. 9), recently shown to control the expression of corneal epithelial stem/progenitor cell and differentiation markers, namely CK15 and CK3^60^. The impact of these mechanotransduction signalling pathways on LESC phenotype merit extensive, in-depth investigation, and should be the subject of future studies along with other potential collagenase-induced effects (e.g., changes in density/affinity of cell adhesion ligands, matrix hydration and composition, and cytoskeleton and nuclear matrix organisation)^17^.

Together, our results provide evidence for the potential of collagenase as a safe corneal treatment to promote and maintain epithelial stem/progenitor cell function. Firstly, they showed that discrete collagenase application allows the modulation of corneal biomechanics without compromising stromal structure and the tissues’ overall shape. This is important, as a significant disruption of the stromal matrix can constitute the basis of several corneal ectatic disorders, including keratoconus^61^. Secondly, collagenase was effective even when applied topically on intact corneas. This advantage obviates the need to remove the epithelium prior to treatment, thus preventing the clinical complications caused by corneal debridement. Thirdly, collagenase treatments failed to elicit any major pro-inflammatory response in vivo or from epithelial cells in vitro. This point is crucial, as severe inflammation of the ocular surface has been associated with corneal injury and disease leading to LESC loss^62^, namely through mechanotransduction pathways^44^. This data also suggested that collagenase treatments could be considered as a suitable adjunct to LESC transplantation in total LSCD cases (e.g., by softening the stromal host tissue), with lower risks of post-op complications due to inflammation. However, further studies will be necessary before this treatment can be used in a clinical ophthalmology setting. In particular, collagenase treatment should be tested on corneal tissues from human LSCD patients (e.g., caused by chemical burns) ex vivo to investigate if increased stiffness varies with injury severity, and understand if adjustments in treatment protocol are required for each individual. In addition, collagenase treatments will have to be evaluated in vivo in longer-term experiments, to determine how enduring the beneficial effects are, and to monitor for any late adverse effects.

In conclusion, this study demonstrates the strong correlation between corneal tissue compliance, YAP-dependent mechanotransduction, and epithelial cell phenotype, using a combination of techniques, such as the non-contact, high-resolution BSM, advanced organ culture, and in vivo modulation of tissue biomechanics. Specifically, our experiments showed that compliant substrates support the growth of undifferentiated LESCs with suppressed YAP signalling, whereas stiffer substrates promoted YAP activation while eliciting cell differentiation, both in vivo and ex vivo. Moreover, we applied this phenomenon to the development of a collagenase-based method to affect LESC phenotype-through-biomechanical modulation both in vivo and ex vivo. This was clearly demonstrated using rabbit corneas, in which stiff areas (normally associated with differentiated cells) were softened and subsequently shown to then support a population of limbal-like undifferentiated cells. The use of enzymes to regulate stem cell phenotype in situ may represent a considerable advance towards developing new therapies based on the modulation of stem/cancer cell mechanotransduction and tissue biomechanics^17,18^. Together with recent advances in line-scanning-BSM^63^ and fluorescence emission-BSM^64^, this work also opens multiple areas of research in mechanobiology, particularly regarding the use of this technology to study the biomechanics of corneal structural features^65^, as well as stem/cancer cells and their corresponding niches within live tissues, in real time, and with unparalleled accuracy, speed, and resolution.

## Methods

### Corneal tissue sourcing

Human corneas analysed by Brillouin spectro-microscopy (BSM) were obtained whole, surrounded by a scleral ring, from the National Disease Research Interchange (NDRI, PA, USA) from cadaveric donors (*n* = 3, ages ranging 42–74 years old). Isolated tissues were kept in dextran-containing Carry-C preservative medium (Alchimia, Italy), in refrigerated conditions during transport, and at room temperature during BSM analysis to maintain the cornea’s natural hydration state, thickness, and transparency. Corneas used for isolation of LESCs or organ culture were kindly provided by Dr. Francisco Figueiredo, Royal Victoria Infirmary, Newcastle-upon-Tyne, UK. A total of 18 corneas were obtained from cadaverous human donors (ages between 27 and 76; average ± S.D. = 47 ± 18 years; male–female donor ratio of 2:3) following informed consent of use for research purposes. All corneas were sourced from donors with no prior history of corneal diseases or ocular trauma and in accordance with the Newcastle University and Newcastle-upon-Tyne Hospital Trust Research Ethics Committees’ guidelines, and were used within 2 weeks of collection.

### Brillouin spectro-microscope setup

The Brillouin spectro-microscope consisted of a confocal microscope coupled to a spectrometer, with a tunable interferometric filter inserted in between to suppress parasitic elastic scattering (Fig. 1a). The single mode illumination (transverse and longitudinal) was provided using a frequency doubled Nd:YVO_4_ diode-pumped solid-state 671 nm laser (CNI, China) operating at 100 mW and coupled into a fibre with a spectral linewidth smaller than 1 MHz, with a 3 mm ⌀ beam collimated output, reflected off a pair of scanning mirrors (Thorlabs, NJ, USA), and expanded to an 8 mm ⌀ by two achromatic doublets (75 and 180 mm focal length) to fill the back focal plane of a ×20 immersion objective (NA = 0.5). A quarter-wave plate inserted just before the objective was used to obtain laser circular polarisation. Scattered light was collected by the same objective, de-scanned by the same mirrors, separated from the excitation light by a polarising beam-splitter, and coupled into a single-mode fibre also acting as confocal pinhole. The elastically scattered light was filtered by destructive interference using a glass prism allowing the passage of inelastically scattered (Brillouin) light, with a piezoelectric actuator to provide a simple feedback mechanism via periodical adjustment of the interferometer angle to produce a 10% modulation in the (post-filtering) elastic amplitude^22^. Light exiting the filter output fibre was collimated to a 0.8 mm ⌀ and focused on the entrance window of a VIPA (Light Machinery, Canada) with 30 GHz free spectral range using a 100 mm cylindrical lens. A 400 mm cylindrical lens imaged the VIPA’s angular spectrum onto a iXon CCD camera (Andor, UK) operated in non-electron-multiplying mode, with the help of a 50 mm cylindrical lens, orientated orthogonal to the others, to provide focusing along the non-dispersed axis.

### BSM imaging and analysis

Imaging of fresh human corneas was performed by submerging tissues in Carry-C medium inside a Petri dish mounted on a vertical translation stage (Newport MFA-CC). The different regions of the cornea were positioned pre-imaging under bright-field, with the corneal limbus identified by the presence of characteristic features such as Palisades of Vogt, focal stromal projections, and pigmented epithelial cells (Supplementary Fig. 1a). By measuring the optical frequency shift of the scattered light, Brillouin scanning can probe the local spontaneous pressure waves within biological tissues, from which the longitudinal modulus can be evaluated^66^. The measured Brillouin shifts were obtained by fitting the Stokes and anti-Stokes components of the spectra with Lorentzian function line profiles using non-linear least squares (SciPy optimised curve fit), and the amplitude of the fitted Lorentzian giving the strength of the spectral signal. The Brillouin shift for the backscatter geometry was given by ${\mathrm{\Delta }}\nu _{\mathrm {B}} = 2/\lambda \sqrt {M/\rho }$, where *λ* is the optical wavelength in the sample, and *M* and *ρ* correspond to the longitudinal modulus and density of the sample, respectively^22^. Relating the hypersonic (GHz) modulus directly to the quasi-static modulus and Young’s modulus is complicated by acoustic dispersion, which can increase moduli significantly at GHz frequencies. Moreover, the effects of sample-induced aberrations can be substantial when imaging deep into the corneal samples, as the point-spread function of the confocal system is rapidly degraded with aberrations, greatly reducing the signal from the focal region. Although the Brillouin shift is not dependent on the signal strength, signal reduction is associated with a relative increase in contributions from regions above and below focus. This results in crosstalk, averaging of the measured Brillouin shift, and degradation of the *z*-sectioning for regions below strong sample inhomogeneity. Such regions are indicated by large reductions (two or more orders of magnitude) in the amplitude of the Lorentzian fitted to the spectrum, relative to that from the immersion medium above the sample, which is unaffected. However, the technique does provide information about variations and changes in mechanical properties at submicron scale^64^, and the imaging of the anterior cornea was consistent with and not impacted by reduced signal strength or aberrations (Supplementary Fig. 1b). Brillouin patterns were also not substantially affected by a possible heterogeneous penetration of Carry-C in the corneal tissue, as significant differences in frequency shift between the corneal sub-epithelium in the centre and the limbus were similarly observed with other immersion media (15 and 13.5 GHz in glycerol; 6.4 and 6.1 GHz in PBS, respectively)^22^.

### Collagen gel production

Plastic-compressed, high-density collagen gels were made by neutralising sterile rat-tail type-I collagen (2.2 mg mL^−1^ in 0.6% acetic acid; First Link Ltd, UK) in 10× modified Eagle’s medium (MEM; Thermo Scientific) and 1 M sodium hydroxide (Sigma-Aldrich) at an 8:1:1 volume ratio. The solution was gently mixed and cast into circular moulds (2 mL per 1.9 cm^2^ ⌀ wells in 12-well plates) prior to gelling for 30 min at 37 °C. Collagen gels were then fully compressed between two layers of nylon mesh (50 µm mesh size) under a fixed load of 134 g for 5 min, or semi-compressed with 64 g for 2.5 min at room temperature, and then coated with laminin (1.5 × 10^−6^ g cm^−2^; Thermo Scientific) for 2 h prior to cell seeding^11,12^. Fully compressed collagen gels were also coated with the matrix extracted from human corneas, to account for the possible influence of natural corneal biochemical cues on cell phenotype. Briefly, stromal tissues isolated from either the central or limbus regions of three human corneas were minced and digested with 1 × 10^4^ activity units L^−1^ of collagenase type-I from *Clostridium histolyticum* (#17018-029, Life Technologies) in PBS for 60 min at 37 °C. Stroma and limbus protein extracts were then precipitated by incubation with 4 × volume of ice-cold ethanol overnight at −20 °C, followed by centrifugation at 10,000 × *g* for 10 min at 4 °C, and resuspended in PBS. The different corneal extracts were drop-spotted onto compressed collagen gels at 5 × 10^−5^ g cm^−2^, and allowed to dry overnight to create a continuous coating.

### Collagenase treatments

Tissue softening was performed by applying sterile collagenase type-I from *C. histolyticum*. Dose and time of incubation were optimised to reproduce the biomechanical and functional properties of semi-compressed collagen gels^13^ or of the human corneal limbus. Briefly, lyophilised collagenase powder was solubilised in phosphate buffered saline (PBS) at 5 × 10^−2^ g L^−1^ (1 × 10^4^ activity units L^−1^) and then applied onto sterilised Whatman Grade 1 filter paper until saturation, i.e., ~0.2 mL cm^−2^ of paper. The collagenase-soaked paper cut-outs were then gently applied onto the surface of dense collagen gels and incubated for up to 60 min at 37 °C. Treatment of whole human corneas was performed by positioning fresh enucleated corneas with their anterior surface up, and then placing collagenase-soaked filter paper cut-outs over half of their central region (Supplementary Fig. 8) for 60 min at 37 °C (2 units cm^−2^ h^−1^ of total collagenase activity, a dose appropriate to consistently soften surfaces to a limbus-like stiffness). The collagenase-soaked filter paper cut-outs were subsequently discarded, and gels or corneas washed thrice with PBS for 15 min on a rocker agitator to remove remaining enzyme and digestion-derived soluble products. PBS-soaked filter paper cut-outs were incubated for 1 h using a similar method, in order to produce mock-treated specimens (treatment time = 0 min). Collagen gels treated for different durations were imaged by bright-field photography using a Nikon DSRL digital camera (Nikon, Japan) immediately after washes, whereas corneas were immersed in Carry-C medium to maintain the cornea’s natural hydration state and then imaged by BSM, or used in organ culture.

### Hydration analysis

Collagen gel weight was measured before treatment (initial weight, *W*_i_) and after treating the entire surface of gels with collagenase for different durations (final weight, *W*_f_). The loss of mass due to collagenase treatment (Δ*W*) was evaluated for each duration of treatment using the equation Δ*W* = *W*_f_/*W*_i_, whereas the hydration of treated gels was evaluated by weighting the collagen gels after desiccation using a benchtop Freeze Dry System (Cole-Palmer, IL, USA). Briefly, gels subjected to different treatments were frozen at −54 °C to allow water sublimation under controlled pressure for 12 h and their dry weight (*W*_d_) measured immediately after. Gel hydration was subsequently calculated as the ratio of (*W*_f_ − *W*_d_)/*W*_f_ and expressed as a percentage. The experiment was performed using 10 independent gels for each duration of treatment (*n* = 10).

### Isolation and culture of corneal limbal epithelial cells

Human LESCs were isolated from the limbus region of a total of nine corneal rings following removal of scleral tissue and the central 7 mm (used for keratectomy). Briefly, ring tissues were dissected into quarters, plated in six-well plates, and subsequently incubated for 5 days in 4 mL of supplemented CnT-7 medium (CellNTec, Switzerland) at 37 °C and 5% CO_2_, to allow the migration and attachment of LESCs onto the culture plate surface while maintaining cell proliferation and extended viability. The corneal tissue was then removed from the plates, and attached cells allowed to proliferate for an additional week, with culture medium change every 2 days. Cell monolayers reaching 70–80% confluence were passaged using Accutase cell detachment solution (Thermo Scientific) for 10 min at 37 °C, and re-plated at 3 × 10^4^ cells cm^2^, up to passage 4, with cells from each donor used separately.

### Organ culture of human corneas

Collagenase-treated whole human corneas were transferred into Transwell culture inserts, washed 3 × 5 min and scraped with an excess of sterile PBS using a rubber-blade cell scraper (Sarstedt) to detach and remove the loosened epithelium, and then seeded with 5 × 10^5^ LESCs in 2 mL of CnT-7 medium. Corneas mock-treated with PBS were used as non-softened controls, using a rigid cell scraper (Corning) to remove the epithelium. The complete removal of epithelial cells was performed and verified in both treated and control conditions using a bright-field microscope. For the ex vivo alkali burn model, human corneas were similarly processed after treatment with strips of filter paper soaked with 0.5 M of NaOH (alkali) or sterile PBS (control) for 60 s, followed by 3 × 5 min washes with an excess of sterile PBS. A subgroup of alkali burn corneas were also subsequently subjected to collagenase treatment, as described. Organ culture was performed at 37 °C and 5% CO_2_ in CnT-7 medium for 2 weeks, followed by a 1-week period in SHEM and air-lifting^13^, with media change every 2 days. The central region of collagenase-treated and control corneas was monitored daily by bright-field microscopy to evaluate re-epithelialisation, and analysed by confocal immunofluorescence microscopy, as described above. Experiments were performed three independent times (*n* = 3), each with cells and corneal tissue from independent donors.

### Confocal immunofluorescence microscopy

Collagen gels were washed in PBS, blocked in PBS supplemented with 5% bovine serum albumin (BSA) and 0.1% Triton X-100 for 1 h, incubated overnight at 4 °C with rabbit anti-collagen type-I primary antibody (Supplementary Table 3) diluted 1:500 in blocking buffer, washed vigorously 4 × 15 min in PBS, and incubated in the dark for 2 h at room temperature with Alexa 594-conjugated goat anti-rabbit secondary antibody (Thermo Scientific) diluted 1:1000 in blocking buffer. After another 4 × 15 min washes with PBS, gels were immersed in Vectashield anti-fade medium (Vector Laboratories, UK), mounted on glass slides, and imaged by confocal fluorescence microscopy using a Nikon A1, with 1 µm-thick optical sections. Fresh human corneal tissues were fixed in 4% paraformaldehyde in PBS for 20 min and then sectioned in thick slices (0.5–0.8 mm) encompassing the central cornea, limbus, and sclera. Individual corneal slices were then blocked in PBS supplemented with 5% BSA and 0.1% Triton X-100 for 3 h, incubated overnight at room temperature with primary antibodies (Supplementary Table 3) diluted 1:500 in blocking buffer, washed 4 × 1 h in PBS, incubated in the dark for 4 h at room temperature with the corresponding secondary antibodies and DAPI (Thermo Scientific) diluted 1:1000 in blocking buffer, washed again 4 × 1 h in PBS, and mounted onto glass slides. Data was analysed using the NIS-Elements and the ImageJ v.1.7 software suite, with quantification of expression performed by evaluating pixel intensity for each independent channel, and normalised against percentage of average pixel intensity of corresponding control. Representative images were taken from each independent gel or corneal tissue sample, for all experiments.

### Atomic force microscopy

Analysis of surface topography of collagen gels treated with collagenase for different durations was performed in static force (contact) mode using an Easyscan 2-controlled FlexAFM atomic force microscope (Nanosurf, Switzerland) equipped with commercial soft contact mode cantilevers (ContAI-G, Budget Sensors, Bulgaria) with a resonant frequency of 13 kHz, and nominal spring constant of 0.2 N/m. Semi-compressed and coated collagen gels were similarly analysed. Briefly, the different collagen gel samples were mounted onto glass slides that were previously covered with a layer of silicone film to avoid the underlying surface to influence measurements, and minimise sample displacement and drift. Surface topography was analysed from three separate regions in each sample, with 512 × two-direction lines scanned at 10 µm s^−1^, 10 nV, and with a P-gain and I-gain of 1. Surface topography maps were line-fitted and represented in false colour with a *z*-scale of 500 nm. The mechanical properties of the collagen gel samples were evaluated by force–distance spectroscopy. The stiffness of the gels was evaluated from 100 force–distance curves acquired at 2 µm s^−1^ from different positions across each sample, and using SPIP data analysis software (Image Metrology A/S, Denmark) for baseline and hysteresis correction, followed by elastic modulus calculation using the Sneddon model, applicable for soft biological materials^67^. Elastic modulus was represented in scatter-dot plots or in terms of frequency, in percentage of measured values, within 0.2 and 1.0 MPa bins. All experiments were performed three independent times (*n* = 3).

### Quantitative immunofluorescence analysis

Collagen gels treated with collagenase across half their surface or in a ring-shape area for 60 min were laminin-coated, seeded with 3 × 10^5^ LESCs, and incubated in CnT-7 medium at 37 °C for up to 4 weeks. Cells were grown on laminin-coated tissue culture plastic for corresponding periods of time, as infinite-stiffness substrate controls. Subsequently, samples were washed twice in PBS, fixed in 4% paraformaldehyde in water for 20 min at room temperature, and washed again with excess PBS. Cells were then permeabilised in PBS containing 0.1% Triton X-100 (PBS-T) for 5 min, blocked with blocking buffer (PBS-T containing 5% BSA) for 1 h, incubated with the rabbit anti-CK3 or anti-CK15 antibodies (Supplementary Table 3) diluted 1:1000 in blocking buffer for 4 h at room temperature, washed again for 3 × 15 min in blocking buffer, and incubated with IRDye 800CW goat anti-rabbit secondary antibody and CellTag 700 stain diluted 1:1000 in blocking buffer for 2 h at room temperature. Samples were finally washed 3 × 15 min in PBS-T before being imaged by near-infrared quantitative analysis using an Odyssey CLx System (Li-Cor Biosciences). Signal specificity was evaluated from samples incubated without either primary or secondary antibodies. The intensity of signal corresponding to cellular CK3 and CK15 detection was determined for the collagenase-treated and untreated areas of the different substrates, and normalised to the number of cells present on the corresponding areas, determined by the CellTag stain signal. Data was represented as protein expression relative to the average expression of untreated (control) areas of the gels, or ratio of CK15/CK3 expression from cells on the different substrates. Experiments were performed three independent times (*n* = 3) using cells from different donors.

### Enzyme-linked immunosorbent assay

Collagen gels treated with collagenase (treated) or PBS (control) for 60 min were laminin-coated, seeded with 3 × 10^5^ LESCs, and incubated in CnT-7 medium at 37 °C for 3 days. Subsequently, culture supernatants were collected on ice, centrifuged at 10,000 × *g* for 10 min at 4 °C, sampled, and analysed for human IL-6 expression using the Quantikine enzyme-linked immunosorbent assay (ELISA) kit (R&D Systems) according to the manufacturer’s instructions. The expression of IL-6 was then normalised for the amount of total protein present in the supernatants, and expressed as pg mL^−1^ µg^−1^ of total protein. Both conditions were assayed three independent times (*n* = 3).

### Quantitative RT-PCR analysis

Collagen gels treated with collagenase (treated) or PBS (control) for 60 min were laminin-coated, seeded with 3 × 10^5^ LESCs, and incubated in CnT-7 medium at 37 °C for up to 1 week. Cells were then harvested using a flexible cell scraper (Sarstedt, Germany), and their mRNA isolated using standard extraction with Trizol (Thermo Scientific). The assessment of mRNA quality was performed using a Nanodrop 2000 spectrophotometer (Thermo Scientific) to ensure the 260/280 ratio was within the 1.8–2.0 range. Synthesis of cDNA from isolated total mRNA was done using the RT2 First Strand kit (Qiagen, Germany) according to the manufacturer’s protocol, in a TcPlus thermocycler (Techne, UK). The polymerase chain reaction (PCR) was carried out using the default thermal profile of the Eco Real-Time System (Illumina, CA, USA), with the following 40 × three-step cycle: 10-s denaturation, 95 °C; 30-s annealing, 60 °C; and 15-s elongation, 72 °C. The transcription levels of *DEFB4* and *KRT3* (differentiation markers), *ABCG2*, *NP63*, *KRT14*, and *NANOG* (limbal markers), and *VEGF*, *IL1A*, *IL1B*, and *IL6* (pro-inflammatory markers) using specific primers (Supplementary Table 4) were calculated by the comparative threshold cycle (CT) (Eco Software v3.1; Illumina), and normalised to the expression of the *POLR2A* housekeeping gene. Data was represented as gene expression relative to that of LESCs grown on stiffer (control) gels, from three independent experiments (*n* = 3), each using cells from independent donors.

### Collagenase treatment of rabbit corneas in vivo

Animal experiments were performed following the appropriate guidelines and after ethical approval from the Institutional Animal Care and Use Committee of the Harry S. Truman Memorial Veterans’ Hospital and the University of Missouri, Columbia, USA. To test the effect of collagenase on the central cornea, 2–3-month-old New Zealand White female rabbits (Covance Research Products) weighing 2.5–3.0 kg were anesthetised and divided into two groups: one with six animals subjected to collagenase treatment in their intact (right eye) and debrided (left-eye) corneas, previously marked in the central region using an 8 mm ⌀ trephine and debrided of the epithelium; and a second group of six animals subjected to mock-treatment. The effect of collagenase on chemically burned limbus tissue was tested using the right eyes of six rabbits subjected to the localised application of 0.5 M NaOH for 25 s (alkali burn) in the temporal side of the limbus, with a sub-group of three animals subsequently receiving collagenase treatment on the burned limbus (treated) (see Supplementary Fig. 11). The corneas from contralateral eyes were used as mock controls. The softening treatment was performed based on the collagenase activity optimised previously in vitro and ex vivo, as it was shown appropriate to produce tissues with limbus-like mechanical properties. Briefly, collagenase was dissolved in 100 µL of pre-warmed 1:19 Dextran–PBS sterile solution (0.2 g L^−1^; 4 × 10^4^ activity units L^−1^), soaked into an Ultracell Corneal Light Shield PVA sponge (Beaver-Visitec, MA, USA), and then applied to the central or limbus region of the cornea for 15 min (2 units cm^−2^ h^−1^ of total collagenase activity). Mock-treatments were performed by applying Dextran–PBS-soaked sponges to the cornea. Corneas were then washed thoroughly by excess saline and evaluated 1 and 5 days (central cornea softening assay) or 0, 2, and 7 days (alkali-burned limbus assay) post-intervention by IOP measurements, unbiased clinical eye examination, and stereo microscopy and slit lamp microscopy. For the central cornea softening assay, six rabbits (three collagenase-treated, three control animals) were sacrificed at each time point after examination, whereas alkali-burned limbus assay animals were sacrificed at day 7, with whole corneas excised, embedded in optical cutting temperature (OCT) medium, and stored at −80 °C until processed for analysis.

### Statistical analysis

In all experiments, error bars represent the standard deviation (S.D.) of the mean, analysed a priori for homogeneity of variance. Replicates from each independent experiment were confirmed to follow a Gaussian distribution, and differences between groups were determined using one-way or two-way analysis of variance (ANOVA) followed by Bonferroni’s multiple comparison post hoc test. Significance between groups was established for *p* < 0.05, *p* < 0.01, and *p* < 0.001, and with a 95% confidence interval. The average ± S.D. Brillouin frequency shift was calculated using 100 points from discrete areas of the corneal tissue (epithelium, sub-epithelial lamina, anterior stroma, sub-limbal conjunctiva) from three independent donors (*n* = 3), and represented as a scatter plot to illustrate intra-tissue variations. The average ± S.D. frequency distribution of elastic modulus of both treated and untreated gels was tested for correlation, and fit to Gaussian curves using a non-linear least-squares regression model, which were then analysed for independence using the *Chi*-square test.

### Reporting Summary

Further information on experimental design is available in the Nature Research Reporting Summary linked to this article.

## Supplementary information

Supplementary information

Supplementary Movie 1

Supplementary Movie 2

Peer Review

Description of Additional Supplementary Files

Reporting Summary

Source data

## Data Availability

The data supporting this study’s findings is available within the paper and its supplementary information files or from the corresponding author on reasonable request. A reporting summary for this article is available as a Supplementary Information file. The source data underlying Figs. 2f, 3c, d, 4c, 5b, c, 6d, e, 7b, 7d, e, and Supplementary Figs. 4c, f, 5a–c, 6b, 7d, and 9 are provided as a Source Data file.
