# Supplementary material for: Assessment of corneal substrate biomechanics and its effect on epithelial stem cell maintenance and differentiation
Source: Nat Commun. 2019 Apr 3;10:1496. doi: 10.1038/s41467-019-09331-6 (PMC6447573; doi:10.1038/s41467-019-09331-6)
Supplement: Supplementary file 1 — Supplementary information [file 41467_2019_9331_MOESM1_ESM.docx]

**Assessment of corneal substrate biomechanics and its effect on epithelial stem cell maintenance and differentiation**

Ricardo M. Gouveia, Guillaume Lepert, Suneel Gupta, Rajiv R. Mohan, Carl Paterson and Che J. Connon

**Supplementary information**

**Supplementary Table 1:** Unbiased clinical eye examination of collagenase- and mock-treated rabbit corneas.

**Supplementary Table 2:** Unbiased clinical eye examination of rabbit corneas subjected to alkali burn with or without subsequent collagenase treatment.

**Supplementary Table 3:** Specific antibodies used to evaluate protein expression by immunohistochemistry.

**Supplementary Table 4:** Specific primer pairs used to evaluate gene transcription by quantitative RT-PCR.

**Supplementary Fig. 1:** Characterisation of the human cornea performed by Brillouin spectro-microscopy (BSM).

**Supplementary Fig. 2:** Composite *X*-*Z* scans of Brillouin frequency shifts of the limbus region of healthy intact human corneas.

**Supplementary Fig. 3:** Mechanical and molecular properties of the limbal niche.

**Supplementary Fig. 4:** Effects of collagenase treatments on collagen gels.

**Supplementary Fig. 5:** Differential cell phenotype on collagenase-treated gels.

**Supplementary Fig. 6:** Expression of differentiation marker CK3 and limbal protein marker CK15 from LESCs grown on an infinite-stiffness substrate.

**Supplementary Fig. 7:** Mechanical and bio-functional properties of corneal matrix-coated and semi-compressed collagen gels.

**Supplementary Fig. 8:** Softening human corneas with collagenase.

**Supplementary Fig. 9:** Quantification of protein markers detected from the confocal immunofluorescence micrographs illustrated in Fig. 3.

**Supplementary Fig. 10:** Effect of collagenase treatment on rabbit corneal vasculature.

**Supplementary Fig. 11:** Rabbit alkali burn model.

**Supplementary Fig. 12:** Effect of collagenase treatment on rabbit corneal vasculature and re-epithelialisation.

**Supplementary Table 1:** Unbiased clinical eye examination of collagenase- and mock-treated rabbit corneas.

| Animal ID | Intervention | Baseline * | | | | | | Post-intervention * | | | | |
| --- | --- | --- | --- | --- | --- | --- | --- | --- | --- | --- | --- | --- |
|  |  | **IOP** | | **Draize** | | **M-S** | | **IOP** | | **Draize** | | **M-S** |
| RM-28 | Collagenase | 12 | 0 | | 0 | | 13 | | 0 | | 0 | |
| RM-29 | Collagenase | 13 | 0 | | 0 | | 12 | | 0 | | 0 | |
| RM-30 | Collagenase | 11 | 0 | | 0 | | 13 | | 0 | | 0 | |
| RM-31 | PBS | 11 | 0 | | 0 | | 13 | | 0 | | 0 | |
| RM-32 | PBS | 13 | 0 | | 0 | | 14 | | 0 | | 0 | |
| RM-33 | PBS | 12 | 0 | | 0 | | 13 | | 0 | | 0 | |
| RM-34 | Collagenase | 13 | 0 | | 0 | | 9 | | 0 | | 0 | |
| RM-35 | Collagenase | 12 | 0 | | 0 | | 9 | | 0 | | 0 | |
| RM-36 | Collagenase | 10 | 0 | | 0 | | 10 | | 0 | | 0 | |
| RM-37 | PBS | 11 | 0 | | 0 | | 8 | | 0 | | 0 | |
| RM-38 | PBS | 13 | 0 | | 0 | | 9 | | 0 | | 0 | |
| RM-39 | PBS | 12 | 0 | | 0 | | 10 | | 0 | | 0 | |

* Values correspond to the animals’ right eye. Softened rabbit corneas showed normal average intraocular pressure (IOP) and no signs of irritation or inflammation prior to intervention (baseline). Corneas treated with either collagenase or PBS (mock-treated) were shown to maintain similar values at both day 1 and 5 post-intervention, with all eyes showing no irritation or inflammation.

**Supplementary Table 2:** Unbiased clinical eye examination of rabbit corneas subjected to alkali burn with or without subsequent collagenase treatment.

| Animal ID | Intervention | Baseline / Day 0 / Day 2 / Day 7* | | | |
| --- | --- | --- | --- | --- | --- |
|  |  | **IOP** | **Fantes** | **Draize** | **M-S** |
| RM-76 | Burn (day 0) | 8 / 10 / 9 / 8 | 0 / 3 / 2 / 2 | 0 / 21 / 21 / 19 | 0 / 3 / 2 / 2 |
| RM-77 | Burn (day 0) | 7 / 9 / 8 / 11 | 0 / 2 / 2 / 1 | 0 / 21 / 21 / 19 | 0 / 3 / 2 / 2 |
| RM-78 | Burn (day 0) | 10 / 10 / 11 / 10 | 0 / 2 / 2 / 1 | 0 / 19 / 21 / 19 | 0 / 3 / 2 / 2 |
| RM-82 | Burn (day 0) + Col (day 2) | 8 / 9 / 10 / 11 | 0 / 2 / 2 / 1 | 0 / 22 / 21 / 19 | 0 / 2 / 2 / 1 |
| RM-83 | Burn (day 0) + Col (day 2) | 9 / 8 / 11 / 10 | 0 / 3 / 2 / 2 | 0 / 23 / 21 / 21 | 0 / 2 / 2 / 1 |
| RM-84 | Burn (day 0) + Col (day 2) | 10 / 9 / 9 / 11 | 0 / 3 / 2 / 2 | 0 / 21 / 21 / 21 | 0 / 2 / 2 / 1 |

***** All animals showed normal average IOP and no signs of irritation or inflammation prior to burn (baseline). Collagenase-treated (burn + col) and alkali burned corneas kept untreated (burn) showed similar values immediately after burn (day 0) and at day 2 and 7 post-burn.

**Supplementary Table 3:** Specific antibodies used to evaluate protein expression by immunohistochemistry.

| Antigen | Host species | Reference | Manufacturer |
| --- | --- | --- | --- |
| ABCG2 | rat | ab24114 | Abcam |
| Beta-catenin | mouse | ab11350 | Abcam |
| CD31 | mouse | 555445 | BD Biosciences |
| Collagen-I | rabbit | ab34710 | Abcam |
| Collagen-I | goat | ab19811 | Abcam |
| Collagen-IV | rabbit | Ab6586 | Abcam |
| Collagen-V | rabbit | ab7046 | Abcam |
| Collagen-VII | rabbit | ab93350 | Abcam |
| Cytokeratin (CK) 3 | mouse | sc-80000 | Santa Cruz Biotechnology |
| Cytokeratin (CK) 3+12 | mouse | ab68260 | Abcam |
| Cytokeratin (CK) 15 | rabbit | ab52816 | Abcam |
| ΔNp63 | rabbit | sc-8343 | Santa Cruz Biotechnology |
| Integrin-α3β1 | mouse | ab24696 | Abcam |
| Integrin-α9 | goat | AF3827 | R&D Systems |
| Laminin-1 | mouse | MA1-21194 | Thermo Scientific |
| Laminin-γ3 | rabbit | STJ93897 | St John's Laboratory |
| VEGFR3 | rabbit | STJ27572 | St John's Laboratory |
| YAP | goat | sc-17141 | Santa Cruz Biotechnology |

**Supplementary Table 4:** Specific primer pairs used to evaluate gene transcription by quantitative RT-PCR.

| Gene | Accession No. | Exon junction | Forward | Reverse |
| --- | --- | --- | --- | --- |
| *ABCG2* | NM_001257386.1 | 7-8 | TCCACTGCTGTGGCATTAAA | CCTGCTTGGAAGGCTCTATG |
| *DEFB4* | NM_004942.2 | 1-2 | TCCTCTTCTCGTTCCTCTTCA | GAGACCACAGGTGCCAATTT |
| *IL1A* | NM_000575.3 | 2-3 | CGGGAAGGTTCTGAAGAAGA | TTTCACATTGCTCAGGAAGC |
| *IL1B* | NM_000576.2 | 1-2 | TCGCCAGTGAAATGATGGCT | TCGGAGATTCGTAGCTGGATG |
| *IL6* | NM_000600.3 | 1-2 | CACACAGACAGCCACTCACC | TTTTCTGCCAGTGCCTCTTT |
| *KRT3* | NM_057088.2 | 8-9 | GAGAGTGTCCGAGTGCTGTC | GCCGTAACCTCCTCCATAGC |
| *KRT14* | NM_000526.4 | 1-2 | CGGCCTGCTGAGATCAAAGA | TCTGCAGAAGGACATTGGCA |
| *NANOG* | NM_024865.2 | 1-2 | CCTGTGATTTGTGGGCCTGA | TGGTGGAAGAATCAGGGCTG |
| *NP63* | NM_003722.4 | 6-7 | TGAGCCACAGTACACGAACC | TGCGCGTGGTCTGTGTTATA |
| *POLR2A* | NM_000937.4 | 8-9 | CATCATCCGAGACAATGGTG | AACAATGTCCCCATCACACA |
| *VEGF* | NM_001025366.2 | 2-3 | CTTGCCTTGCTGCTCTACCT | CCAGGGTCTCGATTGGATGG |

**Supplementary Figures**

**
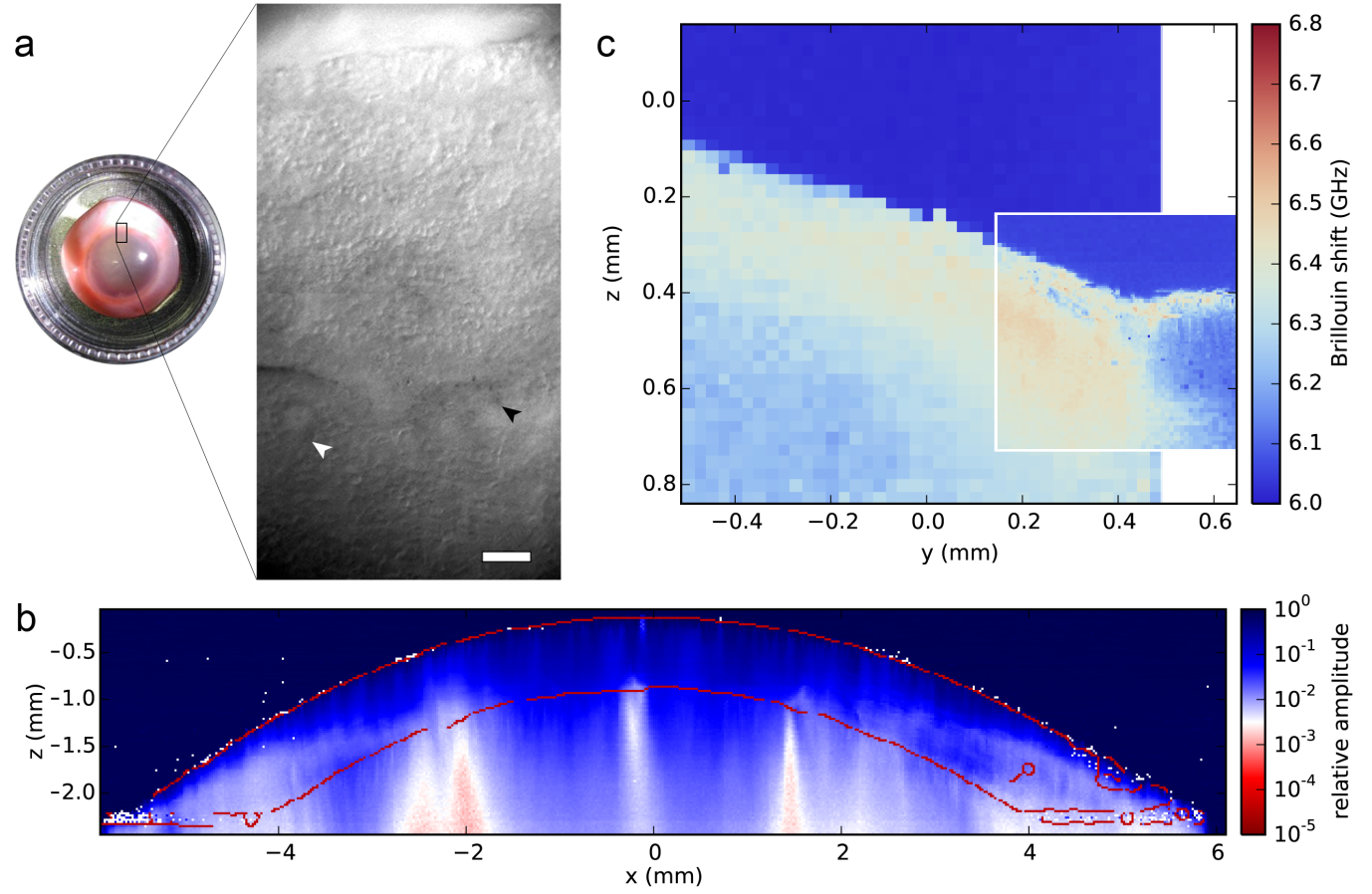
**

**Supplementary Fig. 1: Characterisation of the human cornea performed by Brillouin spectro-microscopy (BSM).**

(**a**) Bright-field micrograph of epithelial cells from the human corneal limbus. The corneal limbus (inset) was localised by the bordering highly-reflective conjunctiva and for presenting characteristic features such as Palisades of Vogt, focal stromal projections (white), and pigmented epithelial cells (black arrowhead) observed at the anterior-most surface of the cornea (scale bar, 100 µm). (**b**) Relative amplitude of the fitted Brillouin peaks for the whole-cornea *X*-*Z* scans in Fig. 1b (with red outline for visual guidance). (**c**) Composite *X*-*Z* scans of Brillouin frequency shifts in the corneal-limbus interface, with distances between measurements of 50 and 5 µm (inset). Image demonstrates that heterogeneity of the mechanical properties of the limbus, where the pockets of low-shift-inducing tissue within the limbal epithelium can only be observed using very high-resolution scans.

**
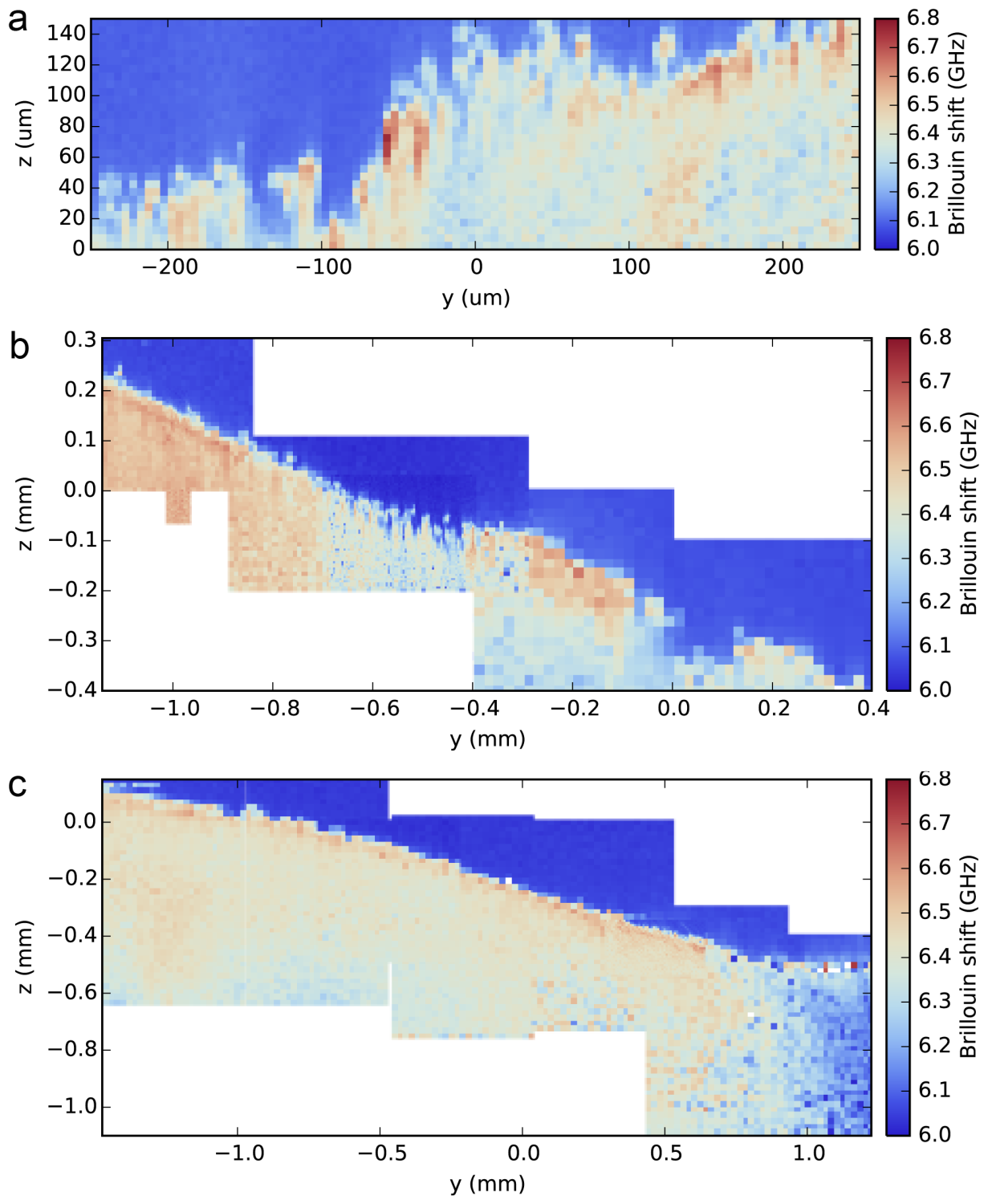
**

**Supplementary Fig. 2: Composite *X*-*Z* scans of Brillouin frequency shifts of the limbus region of healthy intact human corneas.**

(**a**) Superior, (**b**) inferior, and (**c**) nasal sections of the human corneal limbus, analysed in high-resolution scans (5 µm distance between measurements). This detailed analysis evidenced the anatomical features of the limbus from distinct corneal sections, namely the greater abundance of Palisades of Vogt, focal stromal projections, and limbal crypts in the superior and inferior limbus comparted to temporal and nasal side of the cornea.


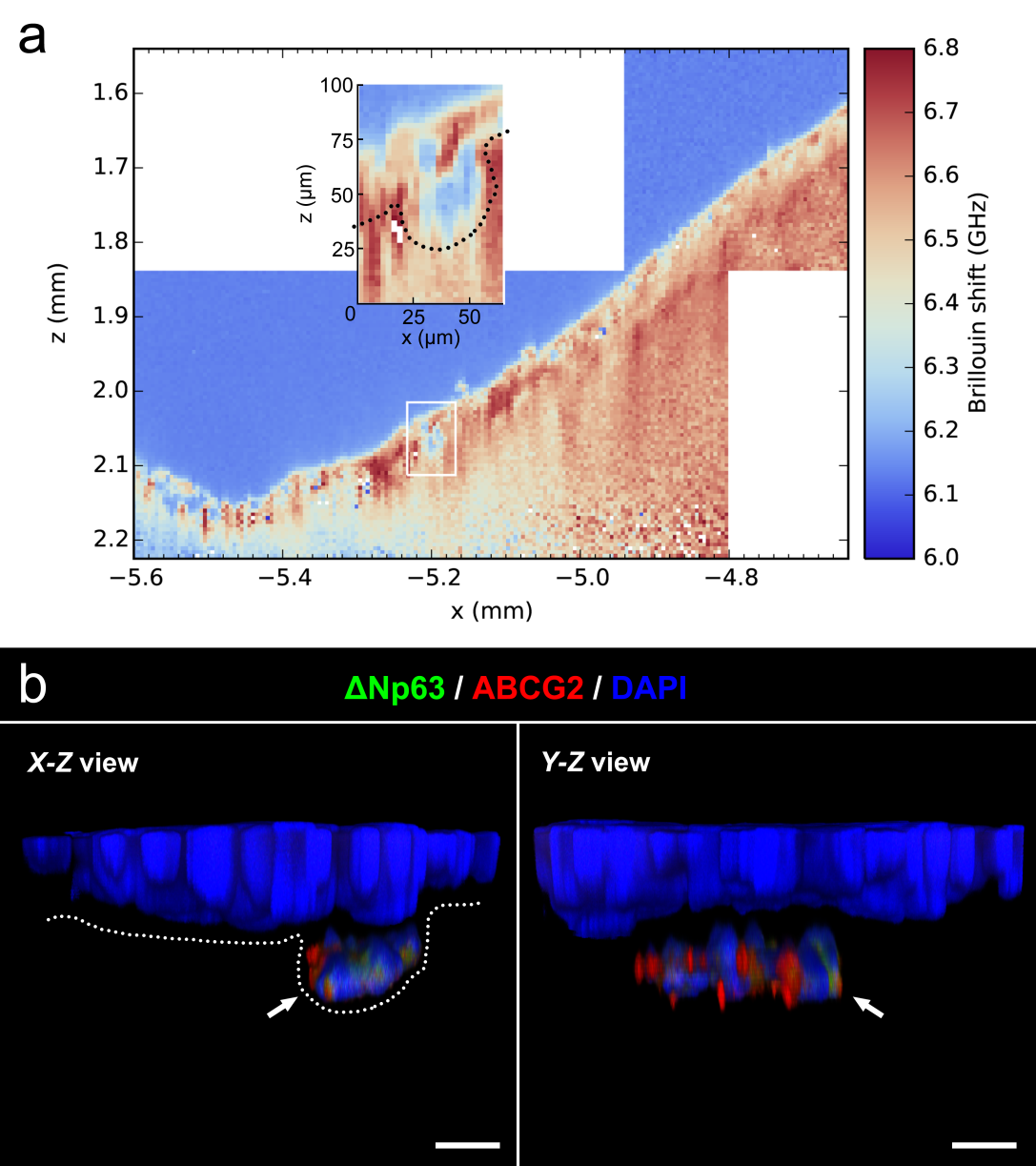


**Supplementary Fig. 3: Mechanical and molecular properties of the limbal niche.**

(**a**) Composite *X*-*Z* scans of Brillouin frequency shifts of the corneal-limbus interface from the temporal section of healthy intact human corneas, with distances between measurements of 5 and 2.5 µm (inset). This detailed analysis evidenced the existence of pockets of lower-frequency shift (black outline) within the limbal epithelium (i.e., containing softer cells and/or matrix), and observed in a 3D reconstruction in Supplementary Movie 2. (**b**) Representative confocal immunofluorescence micrographs (*X-Z* and *Y-Z* views of 3D reconstruction) of pockets within the limbus (white outline), populated by cells (arrows) co-expressing limbal markers ΔNp63 (green) and ABCG2 (red) previously identified within limbal crypts. Nuclei were stained with DAPI (blue). A good shape and size correlation between pockets imaged by Brillouin and immunofluorescence microscopy was observed. Scale bars, 25 µm.

**
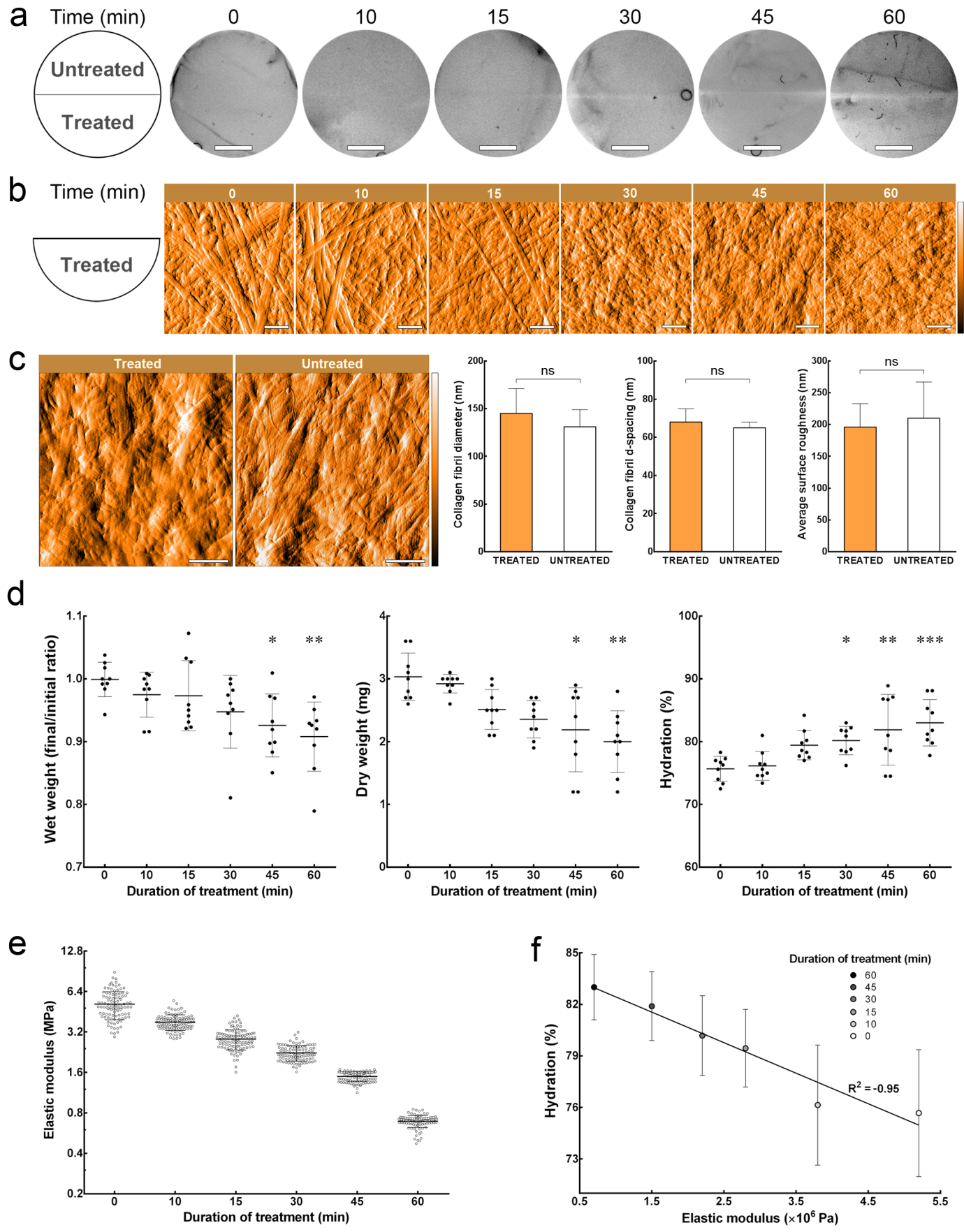
**

**Supplementary Fig. 4: Effects of collagenase treatments on collagen gels.**

(**a**) Bright-field photographs of compressed collagen gels treated with collagenase (0 to 60 min). The softening process resulted in decreased gel density in direct proportion to duration of treatment, as indicated by the increased gel translucence in the treated half-surface area. Scale bars, 5 mm. (**b**) Topography of compressed collagen gels after collagenase treatments analysed by atomic force microscopy (AFM). Representative scans of treated gels showed the clearance of larger collagen fibre bundles with increasing duration of collagenase treatment up to 60 min, with the surface of treated gels showing a higher prevalence of amorphous components. However, both nanostructure of individual collagen fibrils (i.e., diameter and *d*-spacing) and nano-topography (surface roughness) were comparable between treated and untreated gels (**c**), as tested by one-way ANOVA (average ± S.D. of n = 3; ns corresponds to *p* > 0.05). False colour depths, 500 nm. Scale bars, 1 µm. (**d**) Hydration of compressed collagen gels after collagenase treatments. Whole gels were analysed for their wet weight (expressed as the ratio between post- and pre-collagenase treatment weight; left panel) and dry weight post-treatment (central panel) in order to calculate gel hydration (post-collagenase wet minus dry weight/wet weight ratio; right panel). The graphs represent the distribution of measured values and corresponding average (centre line) ± S.D. (whiskers) from 10 independent gels (n = 10; *, **, and *** correspond to *p* < 0.05, 0.01, and 0.001 after one-way ANOVA, respectively). (**e**) Collagen gel stiffness was analysed after collagenase treatments by force-distance spectroscopy using AFM. Data represents corresponding average (centre line) ± S.D. (whiskers) of elastic modulus, *E* (MPa), from three independent experiments (n = 3). (**f**) Correlation between hydration and stiffness of compressed collagen gels after collagenase treatments. The values of hydration and stiffness from collagen gels treated with collagenase for 0-60 min were shown to be inversely correlated, as indicated by the linear regression curve (fitness, R^2^ = -0.95). Source data are provided as a Source Data file.


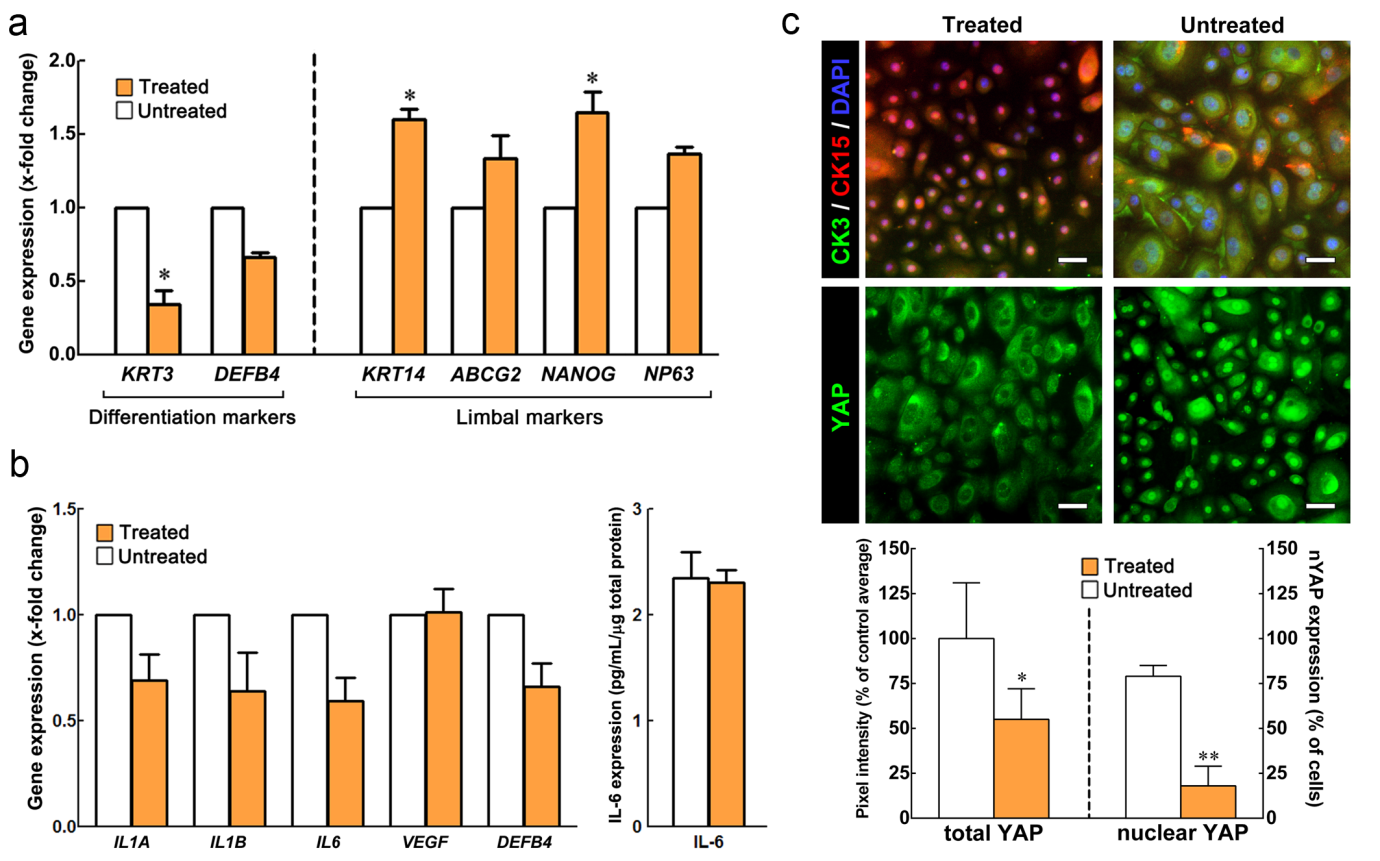


**Supplementary Fig. 5: Differential cell phenotype on collagenase-treated gels.**

The effects of collagenase treatment on gene marker expression were evaluated at the transcriptional level by RT-PCR (**a**-**b**) and protein level by ELISA (**b**) and immunofluorescence analysis (**c**). The expression of (**a**) *KRT3* and *DEFB4* (differentiation markers), and of *KRT14, ABCG2*, *NANOG*, and *NP63* (limbal markers), or of (**b**) pro-inflammatory gene markers was analysed in human limbal epithelial stem cells (LESCs) grown on collagen gels previously treated with collagenase for 60 min (treated; orange bars), normalised to the expression of the housekeeping gene *POLR2B*, and represented as fold-change of the control (cells grown on untreated gels; white bars). IL-6 expression was analysed from culture supernatants by ELISA after normalisation by the amount of total protein present in media (**b**, right panel). (**c**) Representative immunofluorescence micrographs of cells grown on treated and untreated collagen gels expressing differentiation marker CK3 and limbal marker CK15 (top) or YAP (bottom panel), and corresponding total and nuclear YAP quantification. Nuclei were stained with DAPI (blue). A good correlation between cell morphology (shape/size) and marker expression profile was observed. All data corresponds to average ± S.D. from three independent experiments (n = 3; * and ** corresponds to *p* < 0.05 and 0.01 after one-way ANOVA, respectively). Source data are provided as a Source Data file. Scale bars, 25 µm.

**
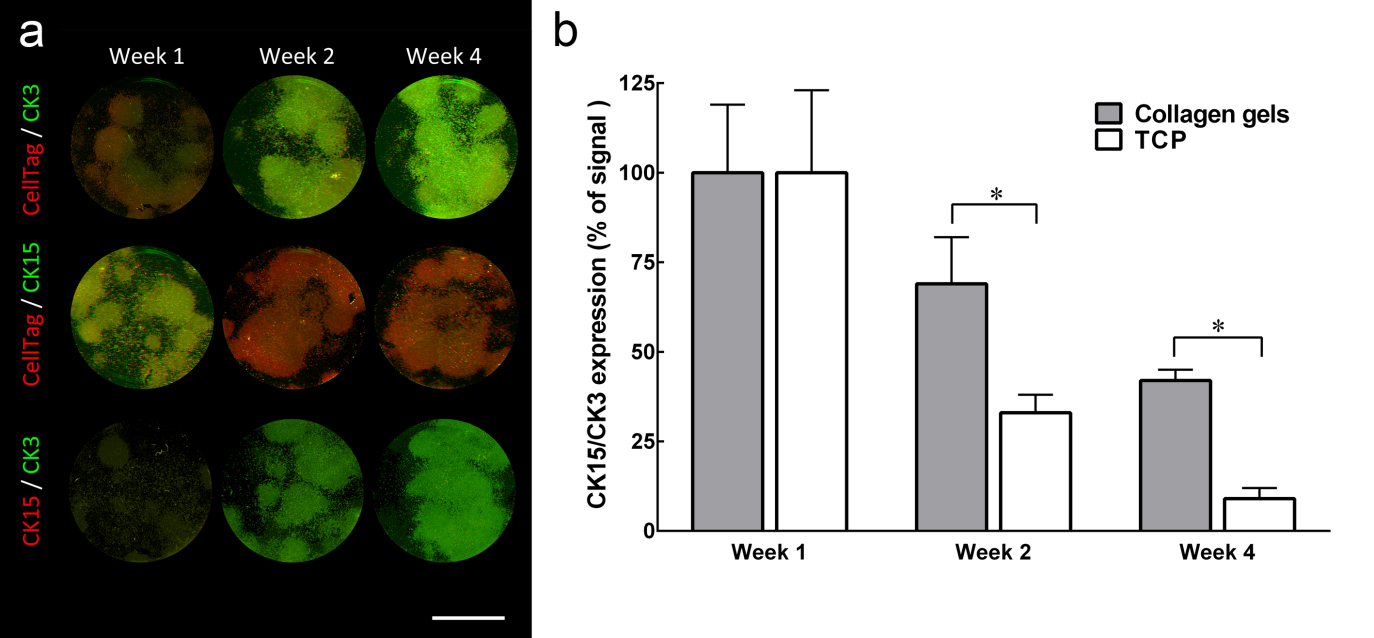
**

**Supplementary Fig. 6: Expression of differentiation marker CK3 and limbal protein marker CK15 from LESCs grown on an infinite-stiffness substrate.**

(**a**) Quantification of CK3 or CK15 (green) and cell number (red staining) after 1, 2, and 4 weeks in culture using infrared fluorescence detection. Scale bar, 10 mm. (**b**) The ratio between CK15 and CK3 expression was calculated after normalisation for total cell number as a measure of LESC phenotype maintenance both on collagen gels (Fig. 3d) and tissue culture plastic (TCP), and represented as the average ± S.D. from three independent experiments (n = 3; * corresponds to *p* < 0.05 after two-way ANOVA). Source data are provided as a Source Data file.


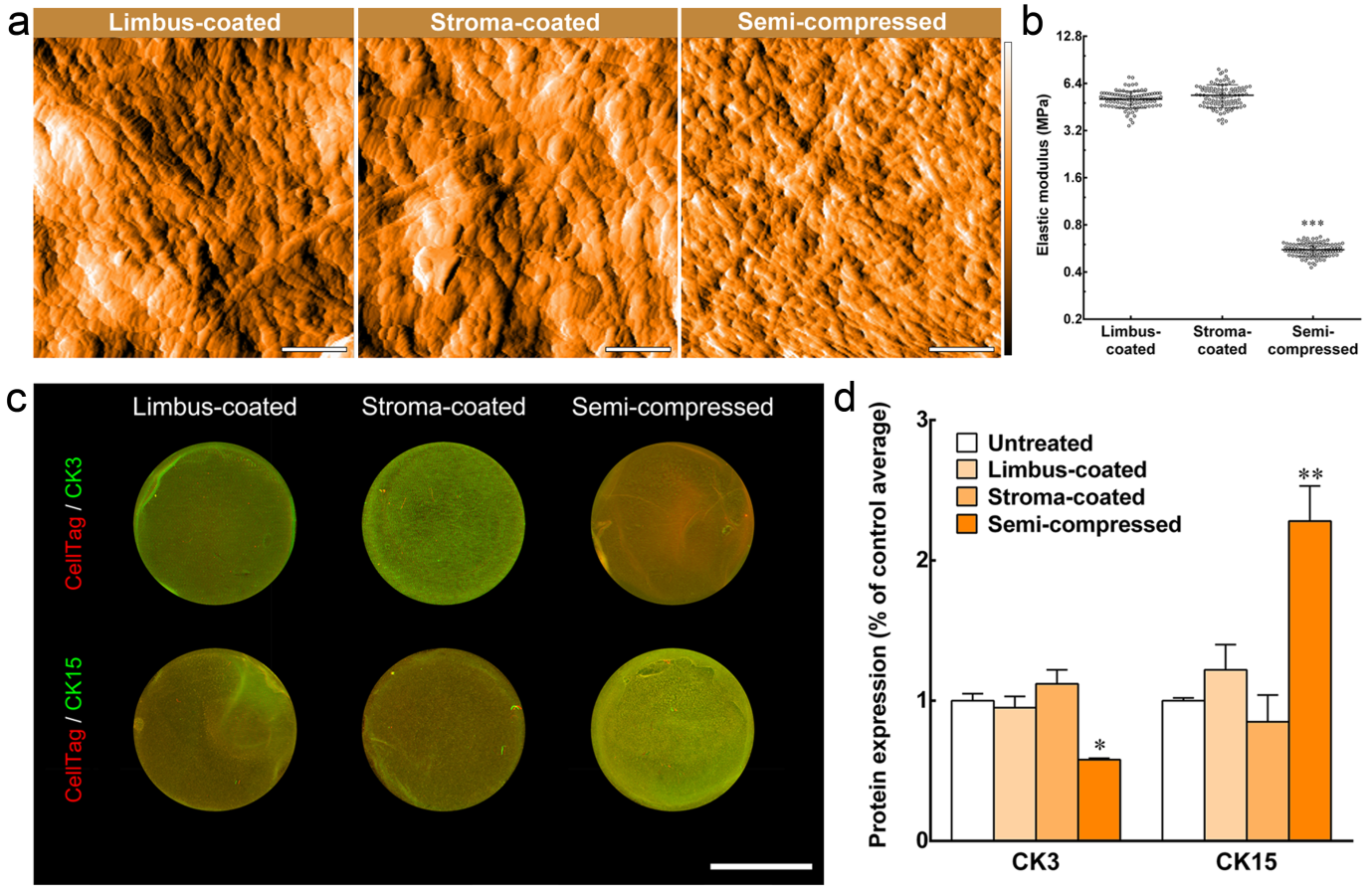


**Supplementary Fig. 7: Mechanical and bio-functional properties of corneal matrix-coated and semi-compressed collagen gels.**

To understand if the modulation of LESC phenotype was due to the softening of the collagen gel matrix, or due to exposure of cryptic signalling motifs resulting from collagenase treatment, LESCs were grown on compressed collagen gels coated with protein extracts obtained from the digestion of corneal stromal tissue or limbus. (**a**) Topography of compressed collagen gels uniformly coated with protein extracts derived from corneal stroma or limbus, and of semi-compressed collagen gels, analysed by atomic force microscopy (AFM). False colour depth, 500 nm; scale bars, 1 µm. (**b**) Force-distance spectroscopy analysis showed that gels coated with the collagenolytic cleavage products of the corneal central stroma or limbus tissues were significantly stiffer than semi-compressed collagen gels, but comparable to uncoated, untreated gels (Fig. 3). The graph represents the distribution of calculated values of elastic modulus, *E* (MPa), and corresponding averages ± S.D. from three independent experiments (n = 3; *** corresponds to *p* < 0.001 after one-way ANOVA). (**c**) Expression of differentiation and limbal protein markers (green) from cells (red staining) grown on coated and semi-compressed collagen gels for 4 weeks in culture, using infrared fluorescence detection. Scale bar, 10 mm. (**d**) Quantification of CK3 and CK15 expression calculated after normalisation for total cell number and represented as the average ± S.D. from three independent experiments (n = 3; * and ** corresponds to *p* < 0.05 and 0.01 after one-way ANOVA, respectively). Source data are provided as a Source Data file. Cells grown on stroma- and limbus-coated gels showed similar levels of CK3 and CK15 expression compared to those on uncoated, untreated gels, but significantly higher CK3 and lower CK15 levels compared to those on semi-compressed or collagenase-softened gels (Fig. 3d). These results further supported the notion that modulation of LESC phenotype only depended upon the mechanical properties of the supporting collagen matrix.


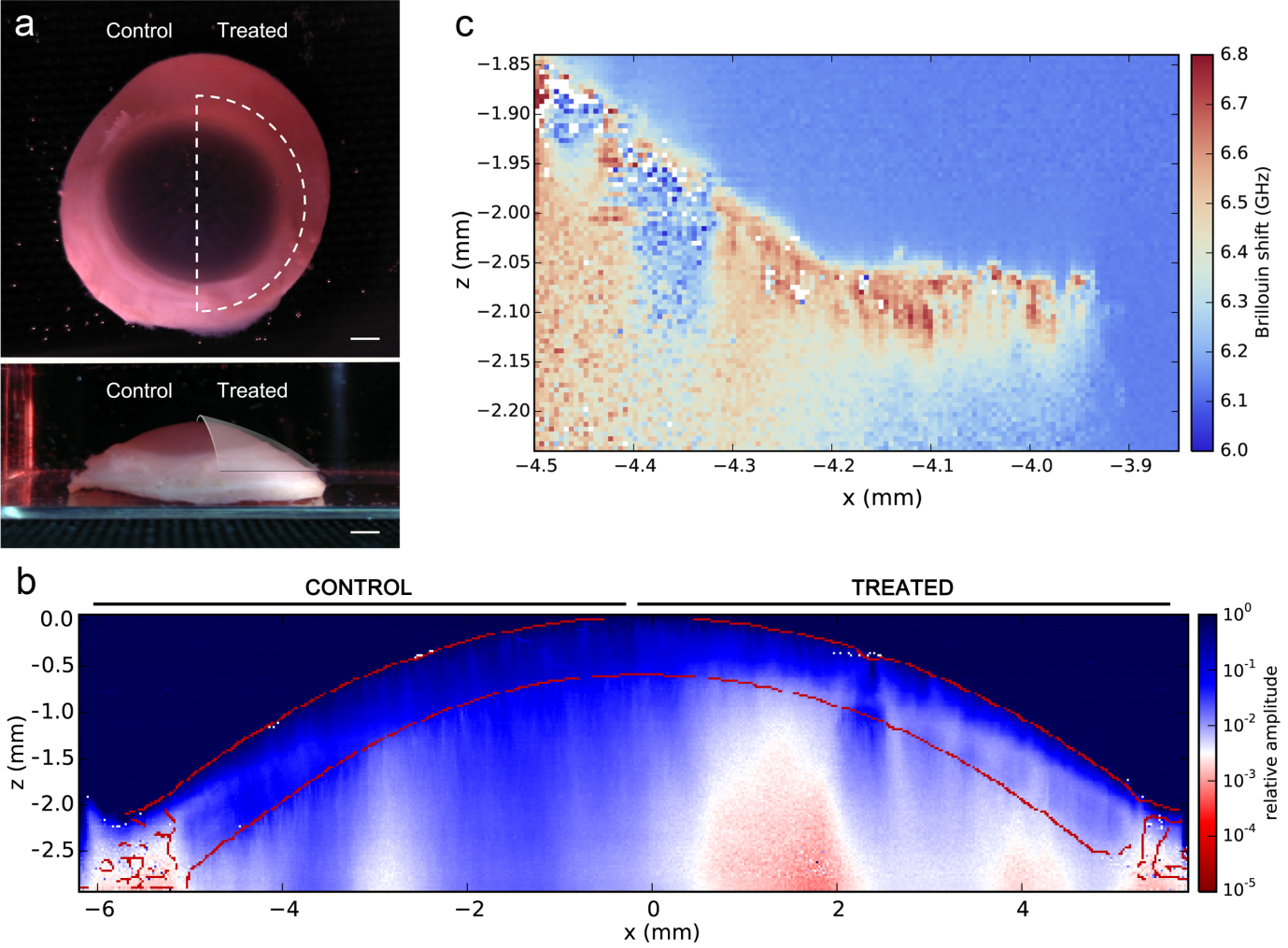


**Supplementary Fig. 8: Softening human corneas with collagenase.**

(**a**) Fresh enucleated corneas were positioned with their anterior surface up, with half of their surface marked for treatment *ex vivo* (top panel, white line). A collagenase-soaked cut-out was placed over the corneal surface (bottom panel) and then removed, followed by extensive washing and imaging by BSM. Scale bars, 1 mm. (**b**) Relative amplitude of the fitted Brillouin peaks for the whole-cornea *X-Z* scans in Fig. 4b (with red outline for visual guidance) showing that the imaging of the anterior cornea after collagenase treatment was consistent, and was not impacted by reduced signal strength or aberrations. (**c**) *X*-*Z* scans of Brillouin frequency shifts of the limbus region after collagenase treatment of human corneas *ex vivo*, with a 5 µm distance between measurements showing that treatment was effective in softening the limbus matrix while maintaining the limbal epithelium intact.


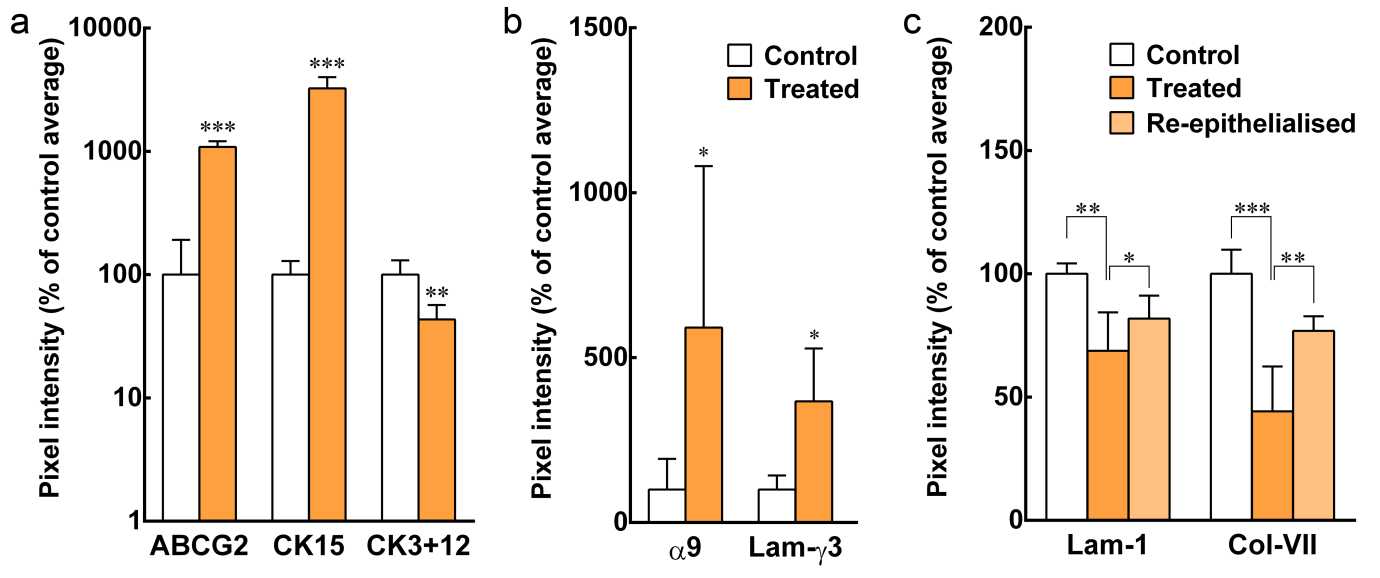


**Supplementary Fig. 9: Quantification of protein markers detected from the confocal immunofluorescence micrographs illustrated in Fig. 3.**

(**a**) Cells repopulating the softened regions of the central cornea (treated) expressed significantly higher levels of limbal markers ABCG2 and CK15, and significantly lower levels of CK3+12 differentiation markers compared to cells growing on the stiffer, untreated central corneas (control). (**b**) Cells on softened corneal matrix also expressed higher levels of limbus-characteristic integrin-α9 and laminin-γ3, and (**c**) were able to deposit laminin-1 and collagen-VII, two basement membrane components that were significantly reduced by collagenase treatment. Expression was represented as average ± S.D. from three independent experiments (n = 3; *, **, and *** corresponds to *p* < 0.05, 0.01, and 0.001 after one-way ANOVA, respectively). Source data are provided as a Source Data file.


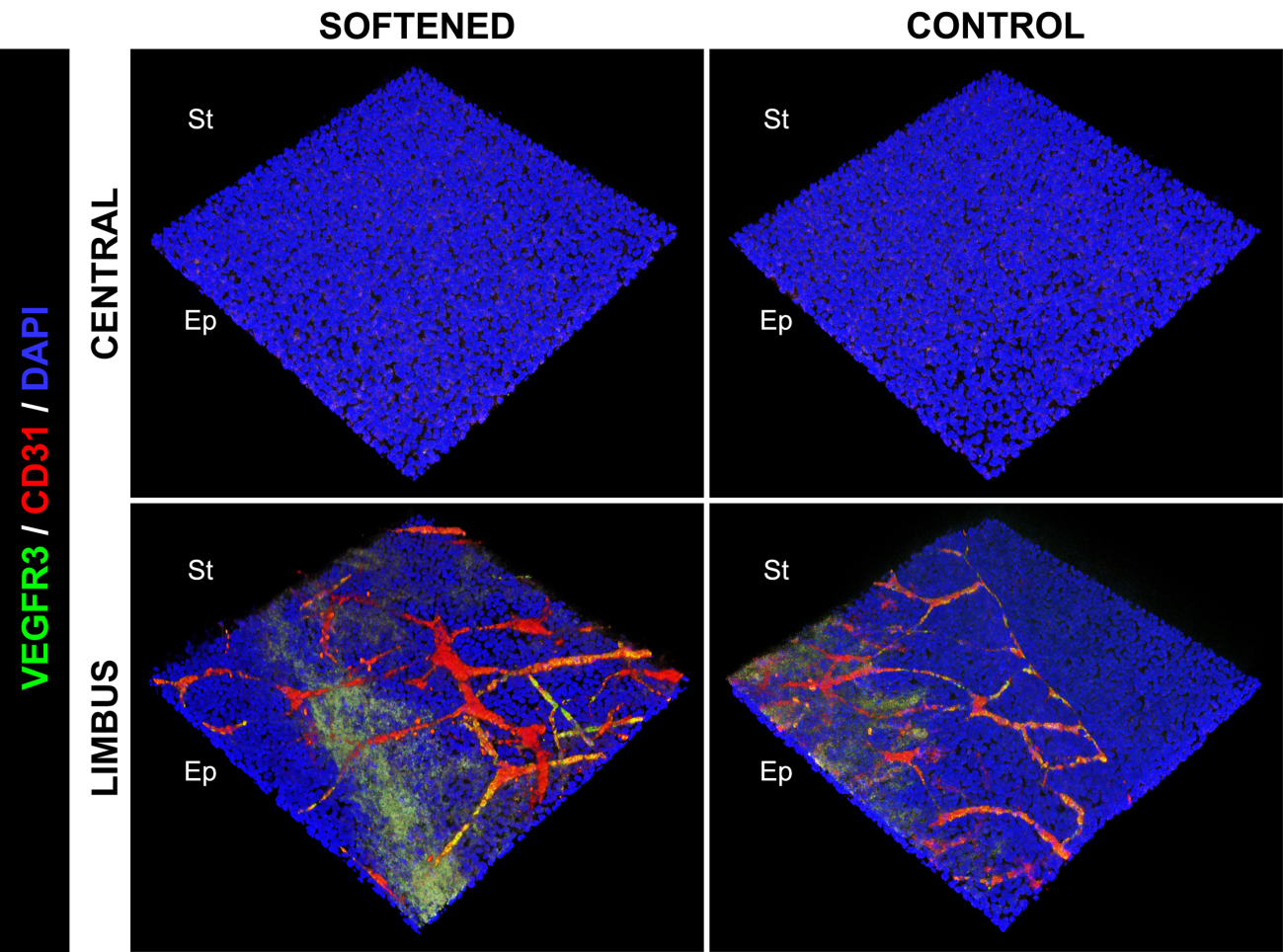


**Supplementary Fig. 10: Effect of collagenase treatment on rabbit corneal vasculature.**

Representative confocal immunofluorescence micrographs (3D reconstruction) were taken 5 days after the central region of intact rabbit corneas was treated with collagenase (softened) or vehicle (control). Vasculature was imaged via identification of VEGFR3 (marker of lymphatic system; green) and CD31 (blood vessel marker; red staining) in a 400 µm square area of both the central cornea and limbus. The position of the corneal stroma (St) and epithelium (Ep) is indicated. Cell nuclei were detected using DAPI.

**
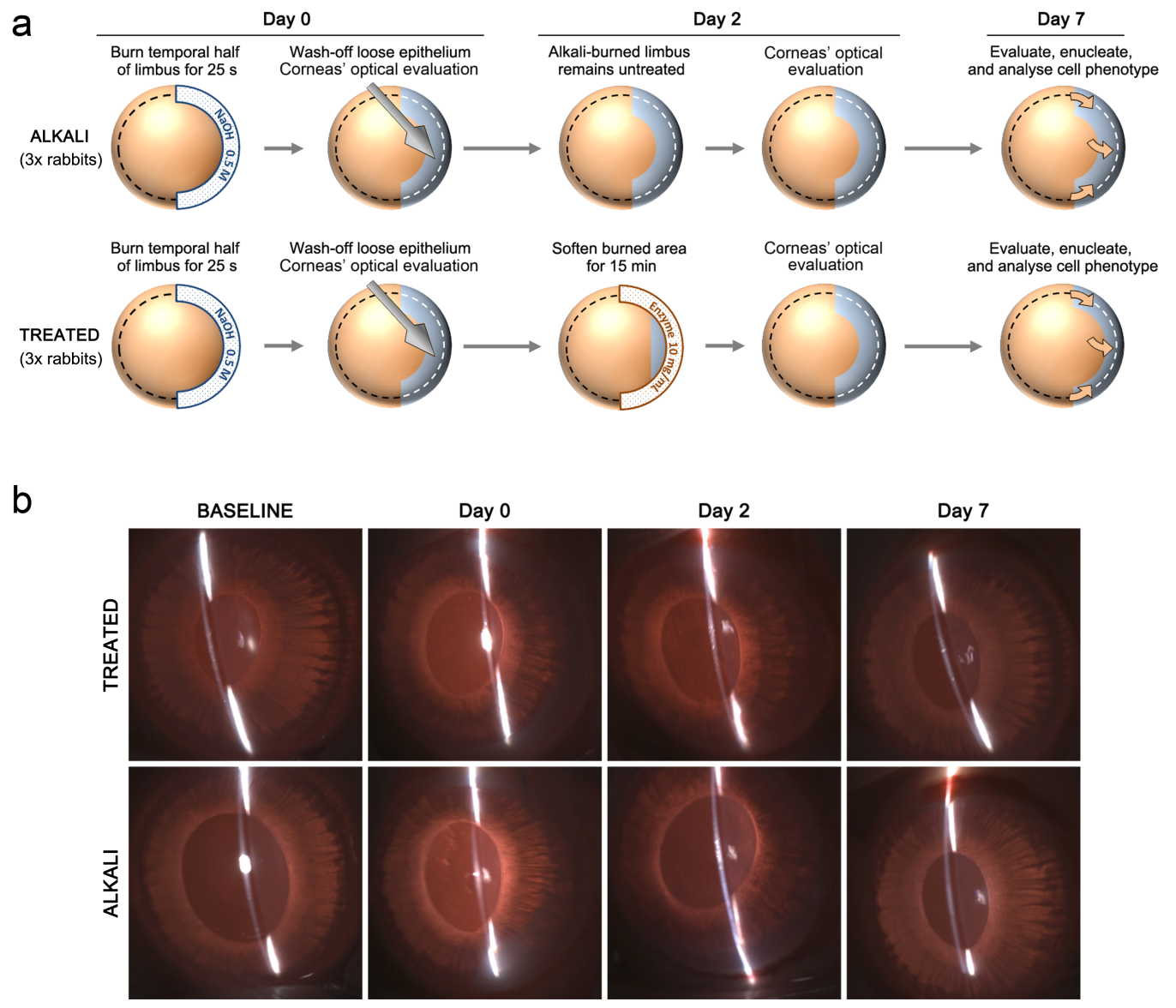
**

**Supplementary Fig. 11: Rabbit alkali burn model.**

(**a**) Schematic representation of the *in vivo* assay. Six rabbits were divided into two subgroups: the first subjected to a chemical burn on the temporal half of the limbus at day 0 (alkali), and the second subjected to a similar burn, which was then treated with collagenase at day 2 (treated). The intact (black) and damaged limbus (white traced lines) is represented, as well as the area affected by the burn (blue) and the predicted sources of re-epithelialisation (yellow arrows). (**b**) Slit-lamp biomicroscopy examination was performed before (baseline) and immediately after burn (day 0), as well as at day 2 and 7 post-burn, with no obvious differences observed between alkali and treated corneas.

**
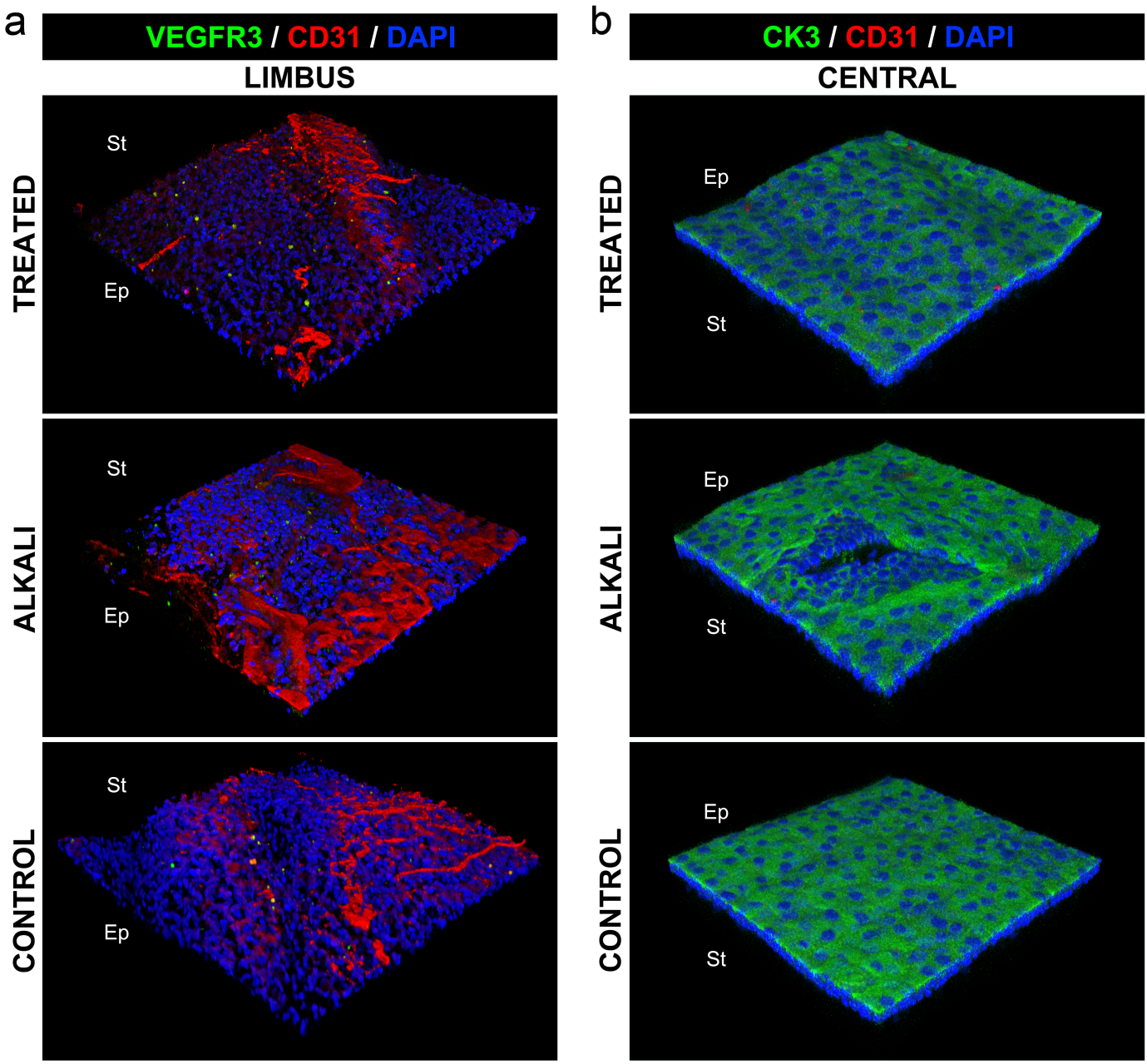
**

**Supplementary Fig. 12: Effect of collagenase treatment on rabbit corneal vasculature and re-epithelialisation.**

Representative confocal immunofluorescence micrographs (3D reconstruction) of 400 µm square area of the limbus (**a**) and central cornea (**b**) were taken 7 days after the limbus of intact rabbit corneas was chemically-burned (alkali) or burned and then treated with collagenase (treated), and then compared to corresponding regions of undamaged rabbit corneas (control). (**a**) Vasculature in the limbus was imaged via identification of VEGFR3 (marker of lymphatic system; green) and CD31 (blood vessel marker; red staining). (**b**) Re-epithelialisation and vasculature in central cornea were imaged via identification of CK3 (differentiated epithelium marker; green) and CD31 (blood vessel marker; red staining), respectively. The position of the corneal stroma (St) and epithelium (Ep) is indicated. Cell nuclei were detected using DAPI. In contrast with burned corneas left untreated, tissues treated with collagenase were able to maintain the central cornea epithelium intact. The loss of epithelium integrity following the limbus burn may have compromised the epithelial barrier function, which in turn may have contributed to the enduring opacity of the untreated (alkali) tissues (Fig. 8).
