# Supplementary material for: Assessment of corneal substrate biomechanics and its effect on epithelial stem cell maintenance and differentiation
Source: Nat Commun. 2019 Apr 3;10:1496. doi: 10.1038/s41467-019-09331-6 (PMC6447573; doi:10.1038/s41467-019-09331-6)
Supplement: Supplementary file 5 — Description of Additional Supplementary Files [file 41467_2019_9331_MOESM5_ESM.docx]

**Supplementary Movie Legends:**

**Title: Supplementary Movie 1**Description: Three-dimensional *X*-*Y*-*Z* scan reconstruction of Brillouin frequency shifts of the limbus region from healthy intact human corneas. Each frame corresponds to a different *X*-*Z* scan, with measurements taken every 5 µm. The heat map colour follows the scheme shown in Fig. 1.

**Title: Supplementary Movie 2**Description: Three-dimensional *X*-*Y*-*Z* scan reconstruction of a low-frequency shift pocket within the limbus of a healthy intact human cornea. Each frame corresponds to a different *X*-*Y* scan, with measurements taken every 2 µm, and with the first frame corresponding to the anterior-most plane. The heat map follows the scheme shown in Supplementary Fig. 2.
