# Supplementary material for: Assessment of corneal substrate biomechanics and its effect on epithelial stem cell maintenance and differentiation
Source: Nat Commun. 2019 Apr 3;10:1496. doi: 10.1038/s41467-019-09331-6 (PMC6447573; doi:10.1038/s41467-019-09331-6)
Supplement: Supplementary file 6 — Reporting Summary [file 41467_2019_9331_MOESM6_ESM.pdf]

nature research

Corresponding author(s): Che Connon

Last updated by author(s): Jan 21, 2019

Reporting Summary

Nature Research wishes to improve the reproducibility of the work that we publish. This form provides structure for consistency and transparency in reporting. For further information on Nature Research policies, see Authors & Referees and the Editorial Policy Checklist.

Statistics

For all statistical analyses, confirm that the following items are present in the figure legend, table legend, main text, or Methods section.

n/a	Confirmed	
☐	☒	The exact sample size (*n*) for each experimental group/condition, given as a discrete number and unit of measurement
☐	☒	A statement on whether measurements were taken from distinct samples or whether the same sample was measured repeatedly
☐	☒	The statistical test(s) used AND whether they are one- or two-sided<br>*Only common tests should be described solely by name; describe more complex techniques in the Methods section.*
☐	☒	A description of all covariates tested
☐	☒	A description of any assumptions or corrections, such as tests of normality and adjustment for multiple comparisons
☐	☒	A full description of the statistical parameters including central tendency (e.g. means) or other basic estimates (e.g. regression coefficient) AND variation (e.g. standard deviation) or associated estimates of uncertainty (e.g. confidence intervals)
☐	☒	For null hypothesis testing, the test statistic (e.g. *F*, *t*, *r*) with confidence intervals, effect sizes, degrees of freedom and *P* value noted<br>*Give P values as exact values whenever suitable.*
☒	☐	For Bayesian analysis, information on the choice of priors and Markov chain Monte Carlo settings
☒	☐	For hierarchical and complex designs, identification of the appropriate level for tests and full reporting of outcomes
☒	☐	Estimates of effect sizes (e.g. Cohen's *d*, Pearson's *r*), indicating how they were calculated

Our web collection on statistics for biologists contains articles on many of the points above.

Software and code

Policy information about availability of computer code

Data collection	Confocal microscopy (NIS-Elements, Nikon, Japan); Brillouin imaging (MicroscoPy™); Near-Infrared fluorescence imaging (Odyssey CLx System, Li-Cor Biosciences, NE, USA)
Data analysis	Confocal microscopy (NIS-Elements, Nikon, Japan; ImageJ v.1.7); AFM analysis (Image Metrology A/S, Denmark); qPCR analysis (Eco Software v3.1; Illumina, CA, USA); Statistical analysis (GraphPad Prism 6.01 and Excel Office 365)

For manuscripts utilizing custom algorithms or software that are central to the research but not yet described in published literature, software must be made available to editors/reviewers. We strongly encourage code deposition in a community repository (e.g. GitHub). See the Nature Research guidelines for submitting code & software for further information.

Data

Policy information about availability of data

All manuscripts must include a data availability statement. This statement should provide the following information, where applicable:

- Accession codes, unique identifiers, or web links for publicly available datasets
- A list of figures that have associated raw data
- A description of any restrictions on data availability

The data that support the findings of this study are available from the corresponding author upon reasonable request.

Field-specific reporting

Please select the one below that is the best fit for your research. If you are not sure, read the appropriate sections before making your selection.

☒ Life sciences ☐ Behavioural & social sciences ☐ Ecological, evolutionary & environmental sciences

For a reference copy of the document with all sections, see nature.com/documents/nr-reporting-summary-flat.pdf

Life sciences study design

All studies must disclose on these points even when the disclosure is negative.

Sample size	Sample sizes determined as per state-of-the-art in the field
Data exclusions	No data was excluded from the analyses
Replication	All attempts at replication were successful
Randomization	Samples allocated randomly
Blinding	Study directors were not blinded to the group allocations, however, the technical staff performing daily animal care and data collection were blinded to group allocations until each study was completed.

Reporting for specific materials, systems and methods

We require information from authors about some types of materials, experimental systems and methods used in many studies. Here, indicate whether each material, system or method listed is relevant to your study. If you are not sure if a list item applies to your research, read the appropriate section before selecting a response.

Materials & experimental systems

n/a	Involved in the study	
☐	☒	Antibodies
☒	☐	Eukaryotic cell lines
☒	☐	Palaeontology
☐	☒	Animals and other organisms
☒	☐	Human research participants
☒	☐	Clinical data

Methods

n/a	Involved in the study	
☒	☐	ChIP-seq
☒	☐	Flow cytometry
☒	☐	MRI-based neuroimaging

Antibodies

Antibodies used	ABCG2 (ab24114, Abcam); Beta-catenin (ab11350, Abcam); CD31 (555445, BD Biosciences); Collagen-I (ab34710, Abcam); Collagen-I (ab19811, Abcam); Collagen-IV (Ab6586, Abcam); Collagen-V (ab7046, Abcam); Collagen-VII (ab93350, Abcam); Cytokeratin 3 (sc-80000, Santa Cruz Biotechnology); Cytokeratin 3+12 (ab68260, Abcam); Cytokeratin 15 (ab52816, Abcam); ΔNp63 (sc-8343, Santa Cruz Biotechnology); Integrin-α3β1 (ab24696, Abcam); Integrin-α9 (AF3827, R&D Systems); Laminin-1 (MA1-21194, Thermo Scientific); Laminin-γ3 (STJ93897, St John's Laboratory); VEGFR3 (STJ27572, St John's Laboratory)
Validation	All antibodies validated, as per manufacturers' description

Animals and other organisms

Policy information about studies involving animals; ARRIVE guidelines recommended for reporting animal research

Laboratory animals	New Zealand White female rabbits two to three month old and weighing 2.5 to 3.0 kg
Wild animals	n/a
Field-collected samples	n/a
Ethics oversight	Ethical approval from the Institutional Animal Care and Use Committee of the Harry S. Truman Memorial Veterans' Hospital and the University of Missouri, Columbia, USA

Note that full information on the approval of the study protocol must also be provided in the manuscript.
