# Supplementary material for: Assessment of corneal substrate biomechanics and its effect on epithelial stem cell maintenance and differentiation
Source: Nat Commun. 2019 Apr 3;10:1496. doi: 10.1038/s41467-019-09331-6 (PMC6447573; doi:10.1038/s41467-019-09331-6)
Supplement: Supplementary file 4 — Peer Review [file 41467_2019_9331_MOESM4_ESM.pdf]

Reviewers' Comments:

Reviewer #1:
Remarks to the Author:
This manuscript presents ex vivo and in vivo experimental results showing the critical role of corneal tissue stiffness on the maintenance / differentiation of limbus epithelial stem cells. Further, it also demonstrates the potential of collagenase-induced tissue softening for treating corneal injury such as alkali burn by normalizing tissue stiffness. The data and interpretation are convincing, and the manuscript is clearly written. The application of Brillouin light scattering microscopy in this study is quite innovative. This manuscript is appropriate for publication in Nature Communications.

Reviewer #2:
Remarks to the Author:
In the manuscript "Modulation of corneal tissue mechanics influences epithelial phenotype" authors use non-contact Brillouin spectro-microscopy to characterize the mechanical properties of human cornea ex vivo as well as effect of substrate stiffness on corneal cell behavior in vitro, ex vivo and in vivo. Most importantly authors represent application of collagenase treatment as a new concept for in situ remodeling of cornea surface with potential clinical applications.

This is definitely an interesting paper regarding the possible applications with collagenase but in its current for it mainly focus and supports the previous knowledge and studies from the field regarding the importance of the surrounding biomechanism for the behavior of corneal/limbal cells. It is well acknowledged in the field, that the elastic properties of the substrate have a role in guiding the differentiation and proliferation of corneal epithelial cells. It is also known that limbal stem cell niche exhibits a very soft environment for the limbal stem cells compared to the surrounding tissue of the cornea and sclera, which both are far more stiff as the limbal area. In order to gain further insight into the actual elastic modulus in corneal limbal niche, an increasing number of studies have been published on measuring the elasticity of the ocular surface. For example, using AFM, Last et al. reported an elastic modulus of around 109 kPa for Bowman's layer of the central cornea, while the anterior basement membrane was found to be much softer, at 7.5 kPa. In the other study by Eberwein et al , the group found an elastic modulus of 10 kPa for the limbus, 19 kPa for the central cornea and 17 kPa for the scleral region. Moers et al. was one of the firsts who found that the elastic modulus has a significant impact on the differentiation of corneal epithelial cells. In their study, cells grown on substrates of physiological stiffness (40 and 80 kPa) maintained a rather early differentiation state represented by the expression of the early differentiation marker cytokeratin 19. However, growth on unphysiologically stiff substrates (1.5 MPa) induced differentiation of the cells represented by the expression of the late differentiation marker cytokeratin 12. Moreover, the cells showed signs of terminal epithelial differentiation. In another study (also cited in the manuscript), using very soft collagen gels with an elastic modulus ranging from 3 Pa (uncompressed) to 2.9 kPa (compressed), Jones et al. observed that corneal epithelial cells exhibited differentiation on the stiffer gels.

In addition, the elastic modulus of the cell culture surface, it is acknowledged that the nanotopography of cellular adhesion also effects the proliferation and differentiation. Based on current understanding this mechanotransduction information is transduced into the cell via the focal adhesion. Via these pathways, integrin-mediated adhesion signals influence stem cell lineage commitment, self-renewal, differentiation and cell migration. To support this, several previous studies have investigated the influence of nanotopography on the behavior of corneal epithelial cells. As an example from in vivo animal studies, Ortega et al. demonstrated that the limbal stem cells cultured on microfabricated rings were able to partially re-epithelialize the corneal surface after destruction of the limabl niche. Also, study by Kang et al has indicated that shear stress plays an important role in cornea and in their elegant study setup intermittent shear flow induced

differentiation, while constant shear flow helped maintaining the stemness of limbal stem cells. This experiment shows that all types of mechanical inputs at the ocular surface must be taken into account, and have implications on the behavior of limbal stem cells as well as more differentiated corneal epithelial cells.

I fully agree with the authors that the possibility to use collagenase for remodeling the surface is highly interesting but unfortunately the methodological approach used in this study, mainly describe the phenomenon happening in vitro, ex vivo and in vivo without any indication of the possible mechanotransduction pathways and molecular cues behind this phenomenon. It would be very important information for the wider community of researchers working in different disciplines. Thus in current form, manuscript is very interesting but preliminary and more depth molecular approaches with suggested mechanisms should be included for high-level paper. I have included some suggestions for the authors that could help strengthening the work and the manuscript.

Abstract:
- In current form, abstract is maybe a bit misleading as BSM was applied for ex vivo corneas and not for live tissue/ in vivo situation thus slide modification/clarification should be made.

Introduction:
- Provide short overview and references in the introduction for the use of collagenase as pharmacological compound for connective tissue softening and a short overview of its tissue compliance in vivo (e.g. inflammation).
- In general, it is very difficult to evaluate the influence of substrate on the behavior of cells in vivo as many other factors not just matrix stiffness is affecting in the limbal niche (e.g stiffness of cells, important part that is not mentioned at all). But anyhow, the use of Brillouin spectro-microscopy for ex vivo tissues is interesting approach. As this technology has been already used for characterization of cornea (cited also by authors), authors should properly cite previous studies in the introduction and highlight importance of their characterization results as compared to those.

Results/discussion:

The results section contains comprehensive/overwhelming amount of in vitro and ex vivo characterization results regarding stiffness and cell behavior greatly supporting previous findings in the field (total of 4 Figure panels and 13 supplementary Figure panels). Instead, the more novel results regarding the applications with collagenase is presented with lesser extend and without mechanistically studies behind cell behavior. Overall, I would suggest that authors concentrate in main result section for the novel findings (collagenase treatment and in vivo modulation) and present data supporting previous findings (material stiffness and hLECs) more depth in supplementary results.

Some details:
- Supplementary Figure3b, are expression of ΔNp63 (green) and ABCG2(red) co-localized in same cells? Please be specific and provide higher resolution images to confirm this.
- Figure 1b, 1c and 1d, is it possible to provide scale bars for the images? It seems that scaling (z(mm)) is not same for Figures 1c and 1d, representative scaling images for both areas would be better for comparison. Figure 1e, with current resolution of the Figure, statistical significances not clearly seen (** and ***), thus please increase quality of the dotplot.
- Data in Figure 3, it is suggested that the ratio between CK3 and CK15 positive cells increase over time, possibly due to the increase in substrate stiffness resulting from cell-driven gel contraction. Could this increased substrate stiffness be confirmed with BSM?
- As demonstrated in Figure 7, alkaline-burned corneas ex vivo failed to maintain the LESC phenotype of repopulating cells and this can be altered with collagenase treatment modification of the substrate stiffness. It would be important that in addition to AFM and FDS, authors could use

BSM to demonstrate that the stiffness of particular whole cornea is changing from the situation before alkaline burn, after alkaline burn and consequently after collagenase treatment. Authors should also discuss if alkaline burn with used method cause systematic stiffness changes or if variability is expected to be high depending on severity of the burn and general variability of stiffness between corneas (e.g. donor age). Also it would be important to at least discuss if in clinical setting variability in injuries would require optimization of collagenase treatment for each and every patient.
- In vivo results (Figure 8), I agree, authors should confirm the absence of conjunctival gobbled cells.
- It is important result that apparently central/partial chemical burn and subsequent collagenase treatment in vivo, maintain cornea integrity and transparency and does not cause inflammation, neo-vascularization or increase IOP during short-term follow-up time. However authors should discuss how approached could be used in limbal traumas e.g chemical burn caused total limbal stem cell deficiency where limbal barrier functions have been compromised and thus possibly causing limitations of the collagenase treatment.
- Most importantly, molecular mechanisms behind cell behavior after collagenase treatment should be studied and confirmed. There is high interests and increased understanding of mechanotransduction pathways in cells including stem cells, thus wide variety of in vitro methods are available for this.

Methods:

- In general, supplementary results contains a lot of important details regarding rationality/performance of the methods, thus authors could consider providing supplementary methods section in addition to supplementary results and then consider carefully what is presented in main methods section (e.g details of important in vivo methods currently only in supplementary data) and what in supplementary methods section. In current form, manuscript is quite difficult to follow.
- Authors could separate own chapter for used tissues/donor corneas with adequate details, currently under chapter BSM. How many donated corneas were used for this study? Please be specific also regarding number of donors of hLECs used in each and every analyses.
- It is widely acknowledged that the elasticity of the human cornea if affected by age, thus it is highly important to consider this variability in any study using cadaveric human corneas. Thus all data should be presented with indication of the age of the donor (raging 42-74) as well as e.g the Brillouin shift of the certain cornea/cornea area before and after the modifications. How much variability there is between corneas from different donor? Apparently BSM measurements where conducted only for 3 corneas (Figure 1e) and those were quite similar but what was the age of donors for these corneas included into the analyses?
- “Compressed collagen gels were coated with matrix extracted from human corneas”, please clarify shortly also in main methods why this approach was needed and clarify if the collagenase digestion in production of this matrix is same as for collagenase use in collagenase treatments. How many corneas was used for production of adequate amount (concentrations?) of this coating matrix?
- It seems that time and dose for the collagenase treatment was determined using semi-compressed collagen gels in vitro. Authors should justify how well that reflects the conditions needed for ex vivo human and in situ rabbit corneas? And again, would they consider that they will be patient to patient variability/need of optimizations in clinical settings?
- Confocal immunofluorescence microscopy: not clear what experiments were performed three independent times? Experiments with semi-compressed collagen gels (data in Figure3)?
- How many corneal rings was used for isolation of corneal limbal epithelial cells? Was cells from each donor used separately or pooled together? Terminology used for cells is not constant in the manuscript both “limbal epithelial stem cells” and “hLECs” used. Authors should clearly indicate the phenotype and characteristics (marker expression) of the cells used in the experiments including passage information.
- Authors should clarify in main methods when cornea extract vs laminin was used for the coating

of collagen gels and why different approach was used.

- Organ culture of whole human corneas. Clarify, how existing epithelium can be removed only with sterile PBS? Is that due to collagenase treatment? How epithelium was removed from untreated corneas? How do you control that all epithelial cells have been removed? How do you distinguish existing/native corneal epithelial cells from seeded hLECS? How many corneas were used for alkaline burn + collagenase treatment? How many as adequate controls in each setting?
- Quatitative immunofluorescence analysis. Marker expression was compared to cells culture on tissue culture plastics. Was that plastic coated with laminin as well? Was same batch of cells plated and used as control as for gels? How many donors or pooled samples of cells used?
- Why ELISA assay for IL-6 was conducted at time point 3 days? That is very early time point as compared to culture times used in other analyses. Why authors performed this analyses in so early phase? Triplicates in ELISA, is that 3 different cell batch or 3 technical replicates?
- Indicated but could be still clarify more carefully, the total number of control rabbits used for collagenase treatment, mock-treatment and alkaline burn +/- treatment?
- It is mentioned that softening treatment was performed based on the collagenase activity optimized previously in vitro and also ex vivo. How many corneas for used for ex vivo optimization and was there a lot of variability between corneas?

Reviewer #3:
Remarks to the Author:
The authors use Brillouin spectro-microscopy, a powerful and reliable technology to evaluate the elastic properties of a material to characterize the mechanical properties of human and rabbit cornea, before and after treatment with collagenase. The main finding of this paper is that the stroma of the limbus (at the junction between the conjunctiva and the cornea) is softer that the corneal stroma and that a collagenase treatment of the cornea results in soften stroma. In response, the corneal basal cells start to express a limbus-like phenotype. The authors conclude that a collagenase treatment may have therapeutic implications for some corneal diseases.

The authors assume that there are no stem cells in the cornea and that stem cells are only located at the limbus. On what grounds do the authors base this claim? The cornea is no different from every other squamous epithelium and is loaded with cells with stem cells functionality (see A. Haddad's paper on rabbit cornea 2014). Corneal basal cells are Tp63-positive in many mammals and the cornea of the rabbit is full of clonogenic cells with extensive growth capability. The results presented in this paper clearly demonstrate that corneal stem cells can fine-tune their gene expression to a differing microenvironment, in this particular case a softer stroma in response to a collagenase treatment. This is an interesting finding. This said this concept has been investigated in the stem cell field for years and it widely now accepted that stiffness and mechanical forces impact stem cell behavior in a variety of tissues and organs.

The paper is a bit long and has too many figures (and certainly too many Supplementary figures…. for instance Supplementary figure 6 is meaningless). Tables 1 (antibodies) and 2 (primers) should be in Supplementary information.

Revision of manuscript NCOMMS-18-17440A-Z

Reply to reviewers' comments

Reviewer #1 (Remarks to the Author):

This manuscript presents ex vivo and in vivo experimental results showing the critical role of corneal tissue stiffness on the maintenance / differentiation of limbus epithelial stem cells. Further, it also demonstrates the potential of collagenase-induced tissue softening for treating corneal injury such as alkali burn by normalizing tissue stiffness. The data and interpretation are convincing, and the manuscript is clearly written. The application of Brillouin light scattering microscopy in this study is quite innovative. This manuscript is appropriate for publication in Nature Communications.

R: We thank the reviewer for these very supporting comments.

Reviewer #2 (Remarks to the Author):

In the manuscript "Modulation of corneal tissue mechanics influences epithelial phenotype" authors use non-contact Brillouin spectro-microscopy to characterize the mechanical properties of human cornea ex vivo as well as effect of substrate stiffness on corneal cell behavior in vitro, ex vivo and in vivo. Most importantly authors represent application of collagenase treatment as a new concept for in situ remodeling of cornea surface with potential clinical applications.

This is definitely an interesting paper regarding the possible applications with collagenase but in its current for it mainly focus and supports the previous knowledge and studies from the field regarding the importance of the surrounding biomechanism for the behavior of corneal/limbal cells. It is well acknowledged in the field, that the elastic properties of the substrate have a role in guiding the differentiation and proliferation of corneal epithelial cells. It is also known that limbal stem cell niche exhibits a very soft environment for the limbal stem cells compared to the surrounding tissue of the cornea and sclera, which both are far more stiff as the limbal area. In order to gain further insight into the actual elastic modulus in corneal limbal niche, an increasing number of studies have been published on measuring the elasticity of the ocular surface. For example, using AFM, Last et al. reported an elastic modulus of around 109 kPa for Bowman's layer of the central cornea, while the anterior basement membrane was found to be much softer, at 7.5 kPa. In the other study by Eberwein et al , the group found an elastic modulus of 10 kPa for the limbus, 19 kPa for the central cornea and 17 kPa for the scleral region. Moers et al. was one of the firsts who found that the elastic modulus has a significant impact on the differentiation of corneal epithelial cells. In their study, cells grown on substrates of physiological stiffness (40 and 80 kPa) maintained a rather early differentiation state represented by the expression of the early differentiation marker cytokeratin 19. However, growth on unphysiologically stiff substrates (1.5 MPa) induced

differentiation of the cells represented by the expression of the late differentiation marker cytokeratin 12. Moreover, the cells showed signs of terminal epithelial differentiation. In another study (also cited in the manuscript), using very soft collagen gels with an elastic modulus ranging from 3 Pa (uncompressed) to 2.9 kPa (compressed), Jones et al. observed that corneal epithelial cells exhibited differentiation on the stiffer gels.

R: As indicated in the introduction, these studies (many of which were included in the original version) indeed comprise the rationale behind our work. However, our findings greatly expand this premise, demonstrating for the first time the influence of corneal biomechanics on epithelial cell phenotype *in vivo*, as well as the ability to modulate cell behaviour via matrix softening. Hence, this study does more than just support previous knowledge, but instead presents novel findings that significantly improve our understanding of corneal biology and regenerative medicine. We have now altered the text to include the additional referred citations and more clearly highlight the innovative parts of our work.

In addition, the elastic modulus of the cell culture surface, it is acknowledged that the nanotopography of cellular adhesion also effects the proliferation and differentiation. Based on current understanding this mechanotransduction information is transduced into the cell via the focal adhesion. Via these pathways, integrin-mediated adhesion signals influence stem cell lineage commitment, self-renewal, differentiation and cell migration. To support this, several previous studies have investigated the influence of nanotopography on the behavior of corneal epithelial cells. As an example from in vivo animal studies, Ortega et al. demonstrated that the limbal stem cells cultured on microfabricated rings were able to partially re-epithelialize the corneal surface after destruction of the limabl niche. Also, study by Kang et al has indicated that shear stress plays an important role in cornea and in their elegant study setup intermittent shear flow induced differentiation, while constant shear flow helped maintaining the stemness of limbal stem cells. This experiment shows that all types of mechanical inputs at the ocular surface must be taken into account, and have implications on the behavior of limbal stem cells as well as more differentiated corneal epithelial cells.

R: We agree that, as indicated by these and other studies, substrate topography (namely the existence of micro-pockets) and shear flow can help define the phenotype of corneal epithelial cells. However, we took these factors into consideration in our experiments. Importantly, we were able to show that tissue topography was not changed by collagenase treatments either *in vitro* (Figure 3, Supplementary Figure 4), *ex vivo* (Figure 4 and 7), or *in vivo* (Figure 5 and 8). In addition, cells were not subjected to varying flow regimens. These results demonstrate that these can be discounted as the main contributing factors for the observed effects on cells.

I fully agree with the authors that the possibility to use collagenase for remodeling the surface is highly interesting but unfortunately the methodological approach used in this study, mainly describe the phenomenon happening in vitro, ex vivo and in vivo without any indication of the possible mechanotransduction pathways and molecular cues behind this phenomenon. It would be very important information for the wider community of researchers working in different disciplines. Thus in current form, manuscript is very interesting but preliminary and more depth molecular

approaches with suggested mechanisms should be included for high-level paper. I have included some suggestions for the authors that could help strengthening the work and the manuscript.

R: We now present new and extensive proof that a YAP-dependent mechanotransduction pathway regulates this phenomenon.

Abstract:

- In current form, abstract is maybe a bit misleading as BSM was applied for ex vivo corneas and not for live tissue/ in vivo situation thus slide modification/clarification should be made.

R: Abstract has now been revised for greater clarity.

Introduction:

- Provide short overview and references in the introduction for the use of collagenase as pharmacological compound for connective tissue softening and a short overview of its tissue compliance in vivo (e.g. inflammation).

R: This information has now been introduced.

- In general, it is very difficult to evaluate the influence of substrate on the behavior of cells in vivo as many other factors not just matrix stiffness is affecting in the limbal niche (e.g stiffness of cells, important part that is not mentioned at all). But anyhow, the use of Brillouin spectro-microscopy for ex vivo tissues is interesting approach. As this technology has been already used for characterization of cornea (cited also by authors), authors should properly cite previous studies in the introduction and highlight importance of their characterization results as compared to those.

R: This information has now been more clearly highlighted.

Results/discussion:

The results section contains comprehensive/overwhelming amount of in vitro and ex vivo characterization results regarding stiffness and cell behavior greatly supporting previous findings in the field (total of 4 Figure panels and 13 supplementary Figure panels). Instead, the more novel results regarding the applications with collagenase is presented with lesser extend and without mechanistically studies behind cell behavior. Overall, I would suggest that authors concentrate in main result section for the novel findings (collagenase treatment and in vivo modulation) and present data supporting previous findings (material stiffness and hLECs) more depth in supplementary results.

R: We understand the reviewer's concern about focusing on the *in vivo* data. However, our extensive *in vitro* and *ex vivo* assays not only represent original findings, but are also necessary for supporting the conclusions from our *in vivo* experiments. We have now increased the focus on the mechanotransduction mechanisms of action.

Some details:

- Supplementary Figure3b, are expression of ΔNp63 (green) and ABCG2 (red) co-localized in same cells? Please be specific and provide higher resolution images to confirm this.

R: Done as suggested.

- Figure 1b, 1c and 1d, is it possible to provide scale bars for the images? It seems that scaling (z(mm)) is not same for Figures 1c and 1d, representative scaling images for both areas would be better for comparison. Figure 1e, with current resolution of the Figure, statistical significances not clearly seen (** and ***), thus please increase quality of the dotplot.

R: Done as suggested.

- Data in Figure 3, it is suggested that the ratio between CK3 and CK15 positive cells increase over time, possibly due to the increase in substrate stiffness resulting from cell-driven gel contraction. Could this increased substrate stiffness be confirmed with BSM?

R: This issue has now been made clearer.

- As demonstrated in Figure 7, alkaline-burned corneas ex vivo failed to maintain the LESC phenotype of repopulating cells and this can be altered with collagenase treatment modification of the substrate stiffness. It would be important that in addition to AFM and FDS, authors could use BSM to demonstrate that the stiffness of particular whole cornea is changing from the situation before alkaline burn, after alkaline burn and consequently after collagenase treatment. Authors should also discuss if alkaline burn with used method cause systematic stiffness changes or if variability is expected to be high depending on severity of the burn and general variability of stiffness between corneas (e.g. donor age). Also it would be important to at least discuss if in clinical setting variability in injuries would require optimization of collagenase treatment for each and every patient.

R: Additional information has now been introduced.

- In vivo results (Figure 8), I agree, authors should confirm the absence of conjunctival gobbled cells.

R: Done as suggested.

- It is important result that apparently central/partial chemical burn and subsequent collagenase treatment in vivo, maintain cornea integrity and transparency and does not cause inflammation, neo-vascularization or increase IOP during short-term follow-up time. However authors should discuss how approached could be used in limbal traumas e.g chemical burn caused total limbal stem

cell deficiency where limbal barrier functions have been compromised and thus possibly causing limitations of the collagenase treatment.

R: This issue has now been discussed.

- Most importantly, molecular mechanisms behind cell behavior after collagenase treatment should be studied and confirmed. There is high interests and increased understanding of mechanotransduction pathways in cells including stem cells, thus wide variety of in vitro methods are available for this.

R: Done as suggested. A considerable amount of new data has now been added to the manuscript clearly showing the impact of collagenase treatment on the expression and intracellular localisation of YAP, a key modulator of cell mechanotransduction. This new data thus provides additional support to our initial claim that the regulation of LESC phenotype by collagenase treatment occurred via modulation of mechanotransduction signalling pathways.

Methods:

- In general, supplementary results contains a lot of important details regarding rationality/performance of the methods, thus authors could consider providing supplementary methods section in addition to supplementary results and then consider carefully what is presented in main methods section (e.g details of important in vivo methods currently only in supplementary data) and what in supplementary methods section. In current form, manuscript is quite difficult to follow.

R: Methods section has been extensively revised.

- Authors could separate own chapter for used tissues/donor corneas with adequate details, currently under chapter BSM. How many donated corneas were used for this study? Please be specific also regarding number of donors of hLECs used in each and every analyses.

R: Done as suggested.

- It is widely acknowledged that the elasticity of the human cornea if affected by age, thus it is highly important to consider this variability in any study using cadaveric human corneas. Thus all data should be presented with indication of the age of the donor (raging 42-74) as well as e.g the Brillouin shift of the certain cornea/cornea area before and after the modifications. How much variability there is between corneas from different donor? Apparently BSM measurements where conducted only for 3 corneas (Figure 1e) and those were quite similar but what was the age of donors for these corneas included into the analyses?

R: This information has now been highlighted.

- “Compressed collagen gels were coated with matrix extracted from human corneas”, please clarify shortly also in main methods why this approach was needed and clarify if the collagenase digestion in production of this matrix is same as for collagenase use in collagenase treatments. How many corneas was used for production of adequate amount (concentrations?) of this coating matrix?

R: This information has now been highlighted.

- It seems that time and dose for the collagenase treatment was determined using semi-compressed collagen gels in vitro. Authors should justify how well that reflects the conditions needed for ex vivo human and in situ rabbit corneas? And again, would they consider that they will be patient to patient variability/need of optimizations in clinical settings?

R: This information has now been added.

- Confocal immunofluorescence microscopy: not clear what experiments were performed three independent times? Experiments with semi-compressed collagen gels (data in Figure3)?

R: This issue has now been clarified.

- How many corneal rings was used for isolation of corneal limbal epithelial cells? Was cells from each donor used separately or pooled together? Terminology used for cells is not constant in the manuscript both “limbal epithelial stem cells” and “hLECs” used. Authors should clearly indicate the phenotype and characteristics (marker expression) of the cells used in the experiments including passage information.

R: Done as suggested.

- Authors should clarify in main methods when cornea extract vs laminin was used for the coating of collagen gels and why different approach was used.

R: Done as suggested.

- Organ culture of whole human corneas. Clarify, how existing epithelium can be removed only with sterile PBS? Is that due to collagenase treatment? How epithelium was removed from untreated corneas? How do you control that all epithelial cells have been removed? How do you distinguish existing/native corneal epithelial cells from seeded hLECS? How many corneas were used for alkaline burn + collagenase treatment? How many as adequate controls in each setting?

R: This information has now been added.

- Quatitative immunofluorescence analysis. Marker expression was compared to cells culture on

tissue culture plastics. Was that plastic coated with laminin as well? Was same batch of cells plated and used as control as for gels? How many donors or pooled samples of cells used?

R: This information has now been added.

- Why ELISA assay for IL-6 was conducted at time point 3 days? That is very early time point as compared to culture times used in other analyses. Why authors performed this analyses in so early phase? Triplicates in ELISA, is that 3 different cell batch or 3 technical replicates?

R: This information has now been added. The earlier time of the analysis followed the instructions in the manufacturer's protocol.

- Indicated but could be still clarify more carefully, the total number of control rabbits used for collagenase treatment, mock-treatment and alkaline burn +/- treatment?

R: Done as suggested.

- It is mentioned that softening treatment was performed based on the collagenase activity optimized previously in vitro and also ex vivo. How many corneas for used for ex vivo optimization and was there a lot of variability between corneas?

R: This information has now been added.

Reviewer #3 (Remarks to the Author):

The authors use Brillouin spectro-microscopy, a powerful and reliable technology to evaluate the elastic properties of a material to characterize the mechanical properties of human and rabbit cornea, before and after treatment with collagenase. The main finding of this paper is that the stroma of the limbus (at the junction between the conjunctiva and the cornea) is softer that the corneal stroma and that a collagenase treatment of the cornea results in soften stroma. In response, the corneal basal cells start to express a limbus-like phenotype. The authors conclude that a collagenase treatment may have therapeutic implications for some corneal diseases.

The authors assume that there are no stem cells in the cornea and that stem cells are only located at the limbus. On what grounds do the authors base this claim? The cornea is no different from every other squamous epithelium and is loaded with cells with stem cells functionality (see A. Haddad's paper on rabbit cornea 2014). Corneal basal cells are Tp63-positive in many mammals and the cornea of the rabbit is full of clonogenic cells with extensive growth capability.

R: For nearly 30 years there has been an overwhelming consensus that, in the human cornea, the epithelial stem/progenitor cells are mainly located in the limbus. This claim is based in numerous widely-cited publications (some of which referenced in the manuscript; please see below*), which clearly demonstrate that limbal epithelial stem cells (LESCs) express a number of molecular markers in addition to Tp63 (e.g. ABCG2, CK14, CK15, α9-integrin). Accordingly, we used several of these markers in our experiments – and not just Tp63 – to accurately identify LESCs and better distinguish them from other transiently-amplifying, clonogenic cell types.

* **Schlotzer-Schrehardt *et al.* (2005) Exp Eye Res, 81: 247** (manuscript reference 34)
Schermer *et al.* (1986) J Cell Biol, 103: 49 (not referenced in manuscript)
Shortt *et al.* (2007) Stem Cells, 25: 1402 (manuscript reference 28)
Li *et al.* (2007) Cell Research, 17: 26 (not referenced in manuscript)
Fatima *et al.* (2008) Eye, 22: 1161 (manuscript reference 51)
Davies *et al.* (2009) Stem Cells, 27: 2781 (manuscript reference 30)
Sun *et al.* (2010) Nature, 463: E10 (not referenced in manuscript)
Yoon *et al.* (2014) World J Stem Cells, 6: 391 (manuscript reference 3)
Di Girolamo *et al.* (2015) Prog Retin Eye Res, 48: 203 (manuscript reference 2)
Nasser *et al.* (2018) Cell Reports, 22: 323 (manuscript reference 58)

The results presented in this paper clearly demonstrate that corneal stem cells can fine-tune their gene expression to a differing microenvironment, in this particular case a softer stroma in response to a collagenase treatment. This is an interesting finding. This said this concept has been investigated in the stem cell field for years and it widely now accepted that stiffness and mechanical forces impact stem cell behavior in a variety of tissues and organs.

R: Unfortunately, the reviewer overlooks the fact that this study provides the first example of an effective enzyme-based method to regulate stem cell phenotype both *in vivo* and *ex vivo*. This concept is, in no way, a widely-known phenomenon, and its great potential impact should not be dismissed so lightly.

The paper is a bit long and has too many figures (and certainly too many Supplementary figures…. for instance Supplementary figure 6 is meaningless). Tables 1 (antibodies) and 2 (primers) should be in Supplementary information.

R: Done as suggested.

END

Reviewers' Comments:

Reviewer #2:
Remarks to the Author:
In this revised version, authors have been able to improve the manuscript substantially and especially the insight for the mechanisms of mechanotransduction is important.
Overall, it would have been helpful for the reviewing process if authors would have provided step-by-step response letter with clear indication of text/chapters, figures etc. modified. Instead authors just stated e.g. “information added”, “done us suggested” without details.
One minor issues that authors could address in the final version of the manuscript:
Authors propose that supp Fig5a represents evidence of a significantly higher clonogenicity (p = 0.048) as compared to cells grown on the stiffer, untreated gels. How authors are able to distinguish that this is true clonogenic activity and not just higher cell proliferation rate? Authors should perform colony forming assay to demonstrate higher clonogenicity. If that has not been done, the texts should be revised accordingly.

Reviewer #4:
Remarks to the Author:
I was asked to comment on whether the authors had sufficiently addressed the comments of reviewer 3, who was no longer available to assess this MS.

Reviewer 3 comment 1:
'The authors assume that there are no stem cells in the cornea and that stem cells are only located at the limbus...'

This comment is based on data presented in a paper by Majo et al (2008) (https://www.ncbi.nlm.nih.gov/pubmed/18830243) who showed that mouse corneal epithelial cells have extremely high regenerative potential over serial rounds of transplantation, and also suggested that limbal epithelial stem cell activity does not contribute to homeostatic maintenance of the uninjured cornea (a role for LESCs would be reserved for after acute wounding). As reviewer 3 states, this leads to a cornel regeneration model very similar to that thought to exist in the skin - a pool of semi-quiescent stem cells required for response to large injury or disease (limbal or hair follicle bulge stem cells), and a population of fast cycling corneal/epidermal basal epithelial keratinocytes doing the day to day work replacing cells lost from the skin or cornea by abrasion and normal turnover. The model is neat and intellectually satisfying, but does not fit all the experimental data obtained by this and other groups (reviewed in Mort et al. (2015; Evaluating alternative stem cell hypotheses for adult corneal epithelial maintenance. World J. Stem Cells 7: 281–299). Multiple lines of evidence have shown that LESCs *do* maintain the uninjured corneal epithelium, and that basal epithelial stem cells, although they have high regenerative potential, are not 'immortal', even over the lifespan of a mouse. The transplantation experiments performed by Majo et al (2008)appear to have been misleading, because they involved surgery that (we now know) modified the normal centripetal patterns of cell migration in the corneal epithelium, and because the corneal epithelial keratinocytes can modify their phenotype in response to their substrate, as described by the authors of the current ms. While there are still questions to address within the field, a reasonable consensus position is that if there are epithelial stem-like cells in the cornea, outside the limbus, their phenotype, turnover and marker expression is different from that in the limbus, and they have never been found. In that respect there is no need for the authors to address this comment further, though they might want to acknowledge the controversy that existed in wake of the Majo et al publication.

Reviewer 3 comment 2:
'The results presented in this paper clearly demonstrate that corneal stem cells can fine-tune their gene expression to a differing microenvironment, in this particular case a softer stroma in

response to a collagenase treatment. This is an interesting finding. This said this concept has been investigated...'

I understand Reviewer 3's point but I feel the comment is a little harsh. Yes there is a substantial literature that uses collagenase to modify wound healing and cell migration in regenerative/biomaterials fields. However I found the authors' in vivo application of the technique and the mechanistic linking to stem cell phenotype, to be novel and quite elegant, and I would not dismiss it lightly. If there were no previous history of modulating substrate compliance to control stem cell phenotype, this paper would be in Nature Nature, not Nature Comms. As it stands, the authors have made a high impact contribution using a pre-existing tool in a new context with new analysis, and I don't think they need to justify this any further.

Revision of manuscript NCOMMS-18-17440A-Z

Reply to reviewers' comments

Reviewer #2 (Remarks to the Author):

In this revised version, authors have been able to improve the manuscript substantially and especially the insight for the mechanisms of mechanotransduction is important.

R: We thank the reviewer for their insightful comments that helped improve the manuscript.

Overall, it would have been helpful for the reviewing process if authors would have provided step-by-step response letter with clear indication of text/chapters, figures etc. modified. Instead authors just stated e.g. "information added", "done us suggested" without details.

R: We apologise for the lack of additional indications, which was mainly due to the somewhat disperse distribution of the changes/additions. In the future, we will strive to specify in detail where each specific change was performed in the manuscript.

One minor issues that authors could address in the final version of the manuscript: Authors propose that supp Fig5a represents evidence of a significantly higher clonogenicity (p = 0.048) as compared to cells grown on the stiffer, untreated gels. How authors are able to distinguish that this is true clonogenic activity and not just higher cell proliferation rate? Authors should perform colony forming assay to demonstrate higher clonogenicity. If that has not been done, the texts should be revised accordingly.

R: This term was applied in reference to the typical clonogenic morphology of cell cultures. We thank the reviewer for pointing out that no colony forming assay was presented, and therefore this should have been referred to as cell proliferation.

Whilst the data did add further support (in a minor way) to our study's conclusions (in terms of cell proliferation), both this and the cell migration assay (previously described in Supplementary Fig. 5a and b) have now been removed from the manuscript to aid in clarification. We took this decision to better focus the discussion upon the effects of substrate compliance on the cells' molecular phenotype.

Reviewer #4 (Remarks to the Author):

I was asked to comment on whether the authors had sufficiently addressed the comments of reviewer 3, who was no longer available to assess this MS.

> Reviewer 3 comment 1: 'The authors assume that there are no stem cells in the cornea and that stem cells are only located at the limbus...'

This comment is based on data presented in a paper by Majo et al. (2008) (https://www.ncbi.nlm.nih.gov/pubmed/18830243) who showed that mouse corneal epithelial cells have extremely high regenerative potential over serial rounds of transplantation, and also suggested

that limbal epithelial stem cell activity does not contribute to homeostatic maintenance of the uninjured cornea (a role for LESCs would be reserved for after acute wounding). As reviewer 3 states, this leads to a cornel regeneration model very similar to that thought to exist in the skin - a pool of semi-quiescent stem cells required for response to large injury or disease (limbal or hair follicle bulge stem cells), and a population of fast cycling corneal/epidermal basal epithelial keratinocytes doing the day to day work replacing cells lost from the skin or cornea by abrasion and normal turnover. The model is neat and intellectually satisfying, but does not fit all the experimental data obtained by this and other groups (reviewed in Mort et al. (2015; Evaluating alternative stem cell hypotheses for adult corneal epithelial maintenance. World J. Stem Cells 7: 281–299). Multiple lines of evidence have shown that LESCs *do* maintain the uninjured corneal epithelium, and that basal epithelial stem cells, although they have high regenerative potential, are not 'immortal', even over the lifespan of a mouse. The transplantation experiments performed by Majo et al. (2008) appear to have been misleading, because they involved surgery that (we now know) modified the normal centripetal patterns of cell migration in the corneal epithelium, and because the corneal epithelial keratinocytes can modify their phenotype in response to their substrate, as described by the authors of the current ms. While there are still questions to address within the field, a reasonable consensus position is that if there are epithelial stem-like cells in the cornea, outside the limbus, their phenotype, turnover and marker expression is different from that in the limbus, and they have never been found. In that respect there is no need for the authors to address this comment further, though they might want to acknowledge the controversy that existed in wake of the Majo et al. publication.

R: We thank the reviewer for the contextualisation and thorough clarification of this subject. We have now revised the manuscript in order to acknowledge this controversy and its subsequent refutation (please see page 3).

> Reviewer 3 comment 2: 'The results presented in this paper clearly demonstrate that corneal stem cells can fine-tune their gene expression to a differing microenvironment, in this particular case a softer stroma in response to a collagenase treatment. This is an interesting finding. This said this concept has been investigated...'

I understand Reviewer 3's point but I feel the comment is a little harsh. Yes there is a substantial literature that uses collagenase to modify wound healing and cell migration in regenerative/biomaterials fields. However I found the authors' in vivo application of the technique and the mechanistic linking to stem cell phenotype, to be novel and quite elegant, and I would not dismiss it lightly. If there were no previous history of modulating substrate compliance to control stem cell phenotype, this paper would be in Nature Nature, not Nature Comms. As it stands, the authors have made a high impact contribution using a pre-existing tool in a new context with new analysis, and I don't think they need to justify this any further.

R: We thank the reviewer for this supportive assessment of our study.

Martin Collinson (University of Aberdeen)

END
